# Integrative revision of the Palaearctic owlfly genus *Deleproctophylla* Lefèbvre (Neuroptera, Myrmeleontidae, Ascalaphinae)

**DOI:** 10.3897/zookeys.1267.173792

**Published:** 2026-01-27

**Authors:** Davide Badano, Yuchen Zheng, Ulrike Aspöck, Horst Aspöck, Roland Dobosz, Rebecca Funari, Roberto Antonio Pantaleoni, Levente Ábrahám, Xingyue Liu

**Affiliations:** 1 Department of Life Sciences, University of Siena, Via Aldo Moro 2, I-53100 Siena, Italy Rippl-Rónai Museum Kaposvár Hungary; 2 National Biodiversity Future Center, Palermo, Italy Research Institute on Terrestrial Ecosystems (IRET), National Research Council of Italy (CNR) Sassari Italy; 3 State Key Laboratory of Animal Biodiversity Conservation and Integrated Pest Management, Institute of Zoology, Chinese Academy of Sciences, Beijing 100101, China University of Sassari Sassari Italy; 4 Department of Entomology, China Agricultural University, Beijing 100193, China University of Siena Siena Italy; 5 Department of Evolutionary Biology, University of Vienna, 1090 Vienna, Austria Institute of Zoology, Chinese Academy of Sciences Beijing China; 6 Zoological Department II, Natural History Museum of Vienna, 1010 Vienna, Austria University of Vienna Vienna Austria; 7 Institute of Specific Prophylaxis and Tropical Medicine, Medical Parasitology, Medical University of Vienna (MUW), 1090 Vienna, Austria China Agricultural University Beijing China; 8 Upper Silesian Museum, Natural History Department, PL. Sobieskiego 2, 41-902 Bytom, Poland Medical University of Vienna (MUW) Vienna Austria; 9 Research Institute on Terrestrial Ecosystems (IRET), National Research Council of Italy (CNR), Sassari, Italy National Biodiversity Future Center Palermo Italy; 10 Department of Agricultural Sciences, University of Sassari, Sassari, Italy Natural History Museum of Vienna Vienna Austria; 11 Rippl-Rónai Museum, Kaposvár, Hungary Upper Silesian Museum Bytom Poland

**Keywords:** Ascalaphinae, barcoding, Central Asia, larva, Mediterranean, new species, species delimitation, taxonomy

## Abstract

The ascalaphid genus *Deleproctophylla* Lefèbvre is a characteristic element of insects from dry, warm grasslands across the Palaearctic, currently comprising five described species distributed in northern Africa, southern Europe, and western Asia. As with other colorful owlfly genera, species of *Deleproctophylla* have traditionally been differentiated based on wing pattern, a trait prone to high variability and misidentification. The genus currently includes five species: *D. australis* (Fabricius), *D. variegata* (Klug), *D. dusmeti* (Navás), *D. gelini* Navás, and *D. bleusei* Kimmins; however, the taxonomic identity of some populations, particularly from Anatolia, has remained uncertain. Even western European species have been affected by taxonomic confusion, as exemplified by *D. bleusei*, whose presence in southern Spain was only recently detected. A comprehensive revision of all species in the genus demonstrated that the shape of the male ectoproct is the most reliable diagnostic character for species identification. This study also led to the discovery of two new species, *D. dandizenor* Badano, Zheng, U. Aspöck & Dobosz, **sp. nov**. from Afghanistan and Pakistan, and *D. tengri* Zheng, Badano, H. Aspöck & Liu, **sp. nov**. from Turkmenistan, Kyrgyzstan, and China, significantly expanding the known range of the genus. Morphological findings were further supported by species delimitation analyses of COI sequences, which helped identify specimens with atypical pigmentation patterns and confirmed the validity of both European species and the newly described *D. tengri***sp. nov**.

## Introduction

The lacewing family Myrmeleontidae can be roughly divided into two distinct morphotypes: antlions (i.e., members of subfamilies Dendroleontinae, Nemoleontinae, Myrmeleontinae, and part of Ascalaphinae), and owlflies (i.e., part of Ascalaphinae), which were traditionally considered separate families (Badano et al. 2017; Machado et al. 2019). Phylogenomic and transcriptomic analyses have confirmed that owlflies (formerly Ascalaphidae) are deeply nested within Myrmeleontidae, supporting a revision of their internal classification and subfamily-level divisions (Winterton et al. 2018; Machado et al. 2019; Vasilikopoulos et al. 2020; Lai et al. 2025). Owlflies (Ascalaphinae) are highly distinctive insects with long-clubbed antennae, large compound eyes, and elongated wings. They are powerful fliers, actively preying on small insects in flight, usually at dusk or night (Tjeder 1992; Oswald and Machado 2018). However, a few, like the two Palaearctic genera—*Libelloides* Schäffer, 1763, and *Deleproctophylla* Lefèbvre, 1842—are one of the few exceptions, being diurnal and most active during the warmest and sunniest hours of the day (H. Aspöck et al. 1980; Tjeder 1992; Michel and Kral 2008). These genera are not only largely sympatric, but often syntopic, particularly in Mediterranean Europe (H. Aspöck et al. 1980). Despite their overlapping distributions, *Libelloides* and *Deleproctophylla* exhibit strikingly different adult morphologies: the former is colorful and conspicuous (with pale yellow or white and black wings), while the latter is buff-colored, with mostly hyaline wings, and well-camouflaged among dry grasses (H. Aspöck et al. 1980). These two genera share very similar male genital structures (H. Aspöck et al. 1980), and their larvae differ only in minor features (Badano and Pantaleoni 2014), despite their phylogenetic affinities has never been tested. The genus *Deleproctophylla* currently comprises five species, i.e., *D. australis* (Fabricius, 1787), *D. variegata* (Klug in Ehrenberg, 1834), *D. dusmeti* (Navás, 1914), *D. gelini* (Navás, 1919) and *D. bleusei* Kimmins, 1949, and is widely distributed from the Mediterranean basin to Central Asia (H. Aspöck et al. 2001). The wing pattern has been traditionally used to distinguish the species of this genus, despite being highly variable, often leading to confusion (Monserrat et al. 2014). This trait has also proven misleading for populations in southern France and Anatolia, where individuals were assigned to *D. australis* (Colombo et al. 2012) based solely on the presence or absence of a small brown spot below the pterostigma in the forewing.

### The entangled taxonomic history of *Deleproctophylla*

Despite *Deleproctophylla* being one of the few ascalaphid genera present in Europe, its taxonomic history is marked by a remarkable series of misinterpretations, confusions, and overlooked species. Fabricius (1787) described *Ascalaphus australis*, the first species of the genus. Later, Klug in Ehrenberg (1834) described *Ascalaphus variegatus* from the Middle East. Lefèbvre (1842) established the genus name *Deleproctophylla*, which Rambur (1842) shortly thereafter emended to *Theleproctophylla*. Hagen (1860) erroneously synonymised *Ascalaphus australis* and *Ascalaphus variegatus* with *Ascalaphus barbarus* (Linnaeus, 1767). This led to the perception of a single *Theleproctophylla* species, *Theleproctophylla barbara*, a view perpetuated by McLachlan (1873) and van der Weele (1904). Van der Weele (1908) reinstated the original spelling *Deleproctophylla*, recognised that *A. barbarus* as belonging to a distinct genus, and validated *D. australis* as a distinct species and the genus type species. However, van der Weele (1908) continued to classify Iberian and French populations as *D. variegata*, primarily based on the absence of a forewing spot, thus considering *D. variegata* to have a widespread distribution. Notably, most taxonomic issues within *Deleproctophylla* center on the French and Iberian species. Navás (1914) further refined the taxonomy by distinguishing the Spanish species from *D. variegata* of Anatolia, describing *D. dusmeti*. However, he overlooked the presence of two distinct *Deleproctophylla* species in Spain, mistaking the second for immature *D. dusmeti*. Later, Kimmins (1949) described *D. bleusei* from North Africa, but the presence of this species in southern Spain was only recognized much later by Monserrat et al. (2014). H. Aspöck et al. (1980), in their influential work on European Neuroptera, also adhered to the single-species concept for the Iberian Peninsula, leading to their description of *D. dusmeti* incorporating diagnostic characters of *D. bleusei*. H. Aspöck et al. (1980) also noted a second morph in Anatolia alongside *D. variegata*, which they termed the ‘dusmeti-like morph’ (‘dusmeti-ähnliches Phänon’). The status of these Anatolian populations remained unclear, and they were variously cited, sometimes even as *D. dusmeti* (Dobosz and Ábrahám 2007). Similarly, records of *D. australis* from southern France, often sympatric with *D. dusmeti*, remained enigmatic (Colombo et al. 2012).

Remarkably, new field sampling and the study of museum collections have revealed two new species, *D. dandizenor* sp. nov. and *D. tengri* sp. nov., considerably expanding the known range of the genus into Asia and reporting it from Afghanistan and China for the first time. The main aims of the present study are: i) to revise the genus *Deleproctophylla* by integrating morphological and molecular data; ii) to test species boundaries using COI-based species delimitation algorithms; and iii) to describe the two new species from Central Asia.

## Materials and methods

### Examined collections

The examined specimens are preserved in the following collections.

**CAU** Entomological Museum, China Agricultural University, Beijing, China

**DBC** Davide Badano Collection, University of Siena, Siena, Italy

**HUAC** Collection of Horst and Ulrike Aspöck, Wien (Vienna), Austria

**MDC** Musée des Confluences, Lyon, France

**MfN** Museum für Naturkunde, Berlin, Germany

**MNHN** Musée National d’Histoire Naturelle, Paris, France

**MSNG** Natural History Museum “Giacomo Doria”, Genova, Italy

**NHMD** Natural History Museum of Denmark, Copenhagen, Denmark

**NHMW** Naturhistorisches Museum, Wien (Vienna), Austria

**NMPC** National Museum of the Czech Republic, Prague, Czech Republic

**RMNH** Naturalis Biodiversity Center, Rijksmuseum van Natuurlijke Historie, Leiden, the Netherlands

**SCM** Rippl-Rónai Museum, Kaposvár, Hungary

**USMB** Upper Silesian Museum, Bytom, Poland

**VM** Victor Monserrat collection, Madrid, Spain

**ZCAU** Yuchen Zheng collection, China Agricultural University, Beijing, China

Locality information was transcribed from collection labels, maintaining the original format. To map the distribution of historical specimens or for those without coordinates, we assigned an approximate point corresponding to the centroid of the named locality on the map.

### Morphological examination

Genitalia were prepared by clearing the abdominal apex in a 20% KOH solution at 100 °C for seven minutes, followed by rinsing with distilled water. The cleared apex was then transferred to glycerin for further examination.

Habitus photographs were taken using a Nikon® D850 digital camera equipped with an AF-S Micro Nikkor 105 mm f/2.8G ED lens and a Zeiss® Axio Zoom.V16 stereo microscope. The head, thorax, and legs were photographed using a Nikon® D850 digital camera with a Laowa® 25mm f/2.8 2.5–5.0× Ultra Macro lens. Images of the pretarsal claws and genitalia were captured with a Leica® DM2000 microscope fitted with a Nikon® D850 digital camera.

The classification system of Myrmeleontidae follows Machado et al. (2019). Wing venation terminology primarily follows Breitkreuz et al. (2017). Morphological terminology follows Tjeder (1992). Genital terminology is based on U. Aspöck and H. Aspöck (2008), due to the controversy regarding the interpretation of genital segments 10–11 in Myrmeleontidae (Marquez-López et al. 2024).

Morphological abbreviations: **C** costa, **Sc** subcosta, **RA** radius anterior, **RP** radius posterior, **MA** media anterior, **MP** media posterior, **CuA** cubitus anterior, **CuP** cubitus posterior, A anal veins, ect ectoproct, **br** branch of ectoproct, **T8** tergite 8, **gp8** gonapophysis 8, **gx8** gonocoxite 8, **T9** tergite 9, **S9** sternite 9, **gx9** gonocoxite 9, **gp9** gonoapophysis 9, **gx11** gonocoxite 11.

### DNA extraction and amplification

Genomic DNA was extracted from the leg of selected specimens, preserved in ethanol (Table 1). Due to the state of preservation of the type specimens, it was not feasible to extract DNA from *D. dandizenor* sp. nov. Mitochondrial DNA fragment Cytochrome Oxidase subunit I was amplified with the forward primer LCO1490 (5’- GGTCAACAAATCATAAAGATATTGG -3’) and the reverse primer HCO2198 (5’- TAAACTTCAGGGTGACCAAAAAATCA -3’) (Folmer et al. 1994). PCRs were executed in 25 μl reaction volume with 2.5 μl of DNA from each sample, 1.25 μl of both forward and reverse primers (10 μM), 2.5 μl of MgCl 2 (25 mM), 2.5 μl of deoxynucleotides (dNTPs, 10 mM), 5 μl of Green GoTaq Flexi Buffer (Promega, US), 0.125 μl of GoTaq Flexi DNA polymerase (Promega, US) [5 u/μl], and 9.875 μl of sterile ddH2O. Amplifications were obtained in a T100 Thermal cycler (BioRad, US) Thermal Cycler, using the following conditions: (i) 95 °C for 5 min; (ii) 35 cycles of 95 °C for 1 min, 50 °C for 1 min, 72 °C for 90 s; (iii) 72 °C for 7 min. The kit Wizard ® SV Gel and PCR Clean-Up System (Promega, US) were employed or PCR products purification, and their DNA concentration was assessed through NanoDrop One (Thermo Scientific, US) device. The quality of PCR results and purifications was checked through 1% agarose gel. Samples were sequenced with the same primers and applying Sanger techniques at the core facility of BMR Genomics (Padua, IT) run on by automated DNA sequencer.

**Table 1.** COI sequences of ascalaphids used to run the analyses.

Accession number	Species	Provenance	Reference
KJ592570.1	*Libelloides coccajus* (Denis & Schiffermüller, 1775)	Germany, Bavaria	(Morinière et al. 2014)
PX674780	*Deleproctophylla bleusei* Kimmins, 1949	Spain, Almería, Cabo de Gata	This study
PX674778	*Deleproctophylla tengri* sp. nov.	China, Xinjiang, Shihezi	This study
PX674779	*Deleproctophylla tengri* sp. nov. [larva]	China, Xinjiang, Shihezi	This study
PX674772	*Deleproctophylla variegata* (Klug in Ehrenberg, 1834)	Greece, Rhodes, Mount Profitis Ilias	This study
PX674785	*Deleproctophylla variegata* (Klug in Ehrenberg, 1834)	Greece, Samos	This study
PX674782	*Deleproctophylla dusmeti* (Navás, 1914)	France, Alpes Maritimes, Villeneuve Loubet	This study
PX674773	*Deleproctophylla dusmeti* (Navás, 1914) “spotted wings”	France, Alpes Maritimes, Villeneuve Loubet	This study
PX674781	*Deleproctophylla dusmeti* (Navás, 1914)	France, Hérault, St. Paul et Valmalle	This study
PX674783	*Deleproctophylla dusmeti* (Navás, 1914)	France, Hérault, St. Paul et Valmalle	This study
PX674777	*Deleproctophylla australis* (Fabricius, 1787)	Italy, Toscana, Nomadelfia	This study
PX674775	*Deleproctophylla australis* (Fabricius, 1787)	Italy, Sardegna, Alghero	This study
PX674774	*Deleproctophylla australis* (Fabricius, 1787)	Italy, Sicily, Agrigento	This study
PX674776	*Deleproctophylla australis* (Fabricius, 1787)	Greece, Crete, Hersonissos	This study
PX674786	*Deleproctophylla australis* (Fabricius, 1787)	Greece, Peloponnese	This study
PX674784	*Deleproctophylla gelini* (Navás, 1919)	Morocco	This study
MT621185.1	“*Deleproctophylla variegata* (Klug in Ehrenberg, 1834)”	Azerbaijan	(Kerimova et al. 2022)
BoldSystems: BGE_00655_A06	*Deleproctophylla australis* (Fabricius, 1787)	Georgia, Tibilisi	BoldSystems
BoldSystems: BGE_00655_A07	*Deleproctophylla australis* (Fabricius, 1787)	Georgia, Tibilisi	BoldSystems
BoldSystems: BGE_00656_B06	*Deleproctophylla australis* (Fabricius, 1787)	Georgia, Tibilisi	BoldSystems

The obtained COI sequence was edited and checked with MEGA XI. The barcode sequence was analyzed through the integrated bioinformatics platform Barcode of Life Data (BOLD) System to test the morphology-based species identification. COI sequences obtained from GenBank were aligned using the ClustalW program implemented in MEGA XI (Table 1).

### Phylogenetic and species delimitation analyses

A Bayesian Inference (BI) analysis was performed to reconstruct the phylogenetic relationships among taxa based on aligned COI sequences using MrBayes 3.2.7. *Libelloides coccajus* (Denis & Schiffermüller, 1775) was selected as an outgroup, based on the potential affinities of these two genera. The analysis was run enforcing the invgamma model. Four Markov chain Monte Carlo (MCMC) chains, of which one was cold and three heated, were run for 1,000,000 generations, setting a burn-in fraction of 50% and sampling the chains every 1000 generations. The convergence of independent runs was assessed through the average standard deviation of split frequencies (<0.01) and potential scale reduction factors (approaching 1).

To test species boundaries among morphologically similar taxa and to support the description of the new species, two single-locus species delimitation approaches were applied: Assemble Species by Automatic Partitioning (ASAP) (Puillandre et al. 2021) and Multi-rate Poisson Tree Processes (mPTP) (Kapli et al. 2017). ASAP is a distance-based method, calculating pairwise genetic distances and assigning a score to different partitions, selecting the most supported one. The analysis was performed on the ASAP web server, using Kimura (K80) transition/transversion (ts/tv) ratio as the nucleotide substitution model. Instead, mPTP is tree-based method, which infers species boundaries by performing Markov Chain Monte Carlo (MCMC) sampling on the supplied phylogenetic tree. The analysis was conducted on the mPTP web server using default parameters with the BI tree as input.

## Results

### Phylogenetic analyses and species delimitation

**Figure 1.**
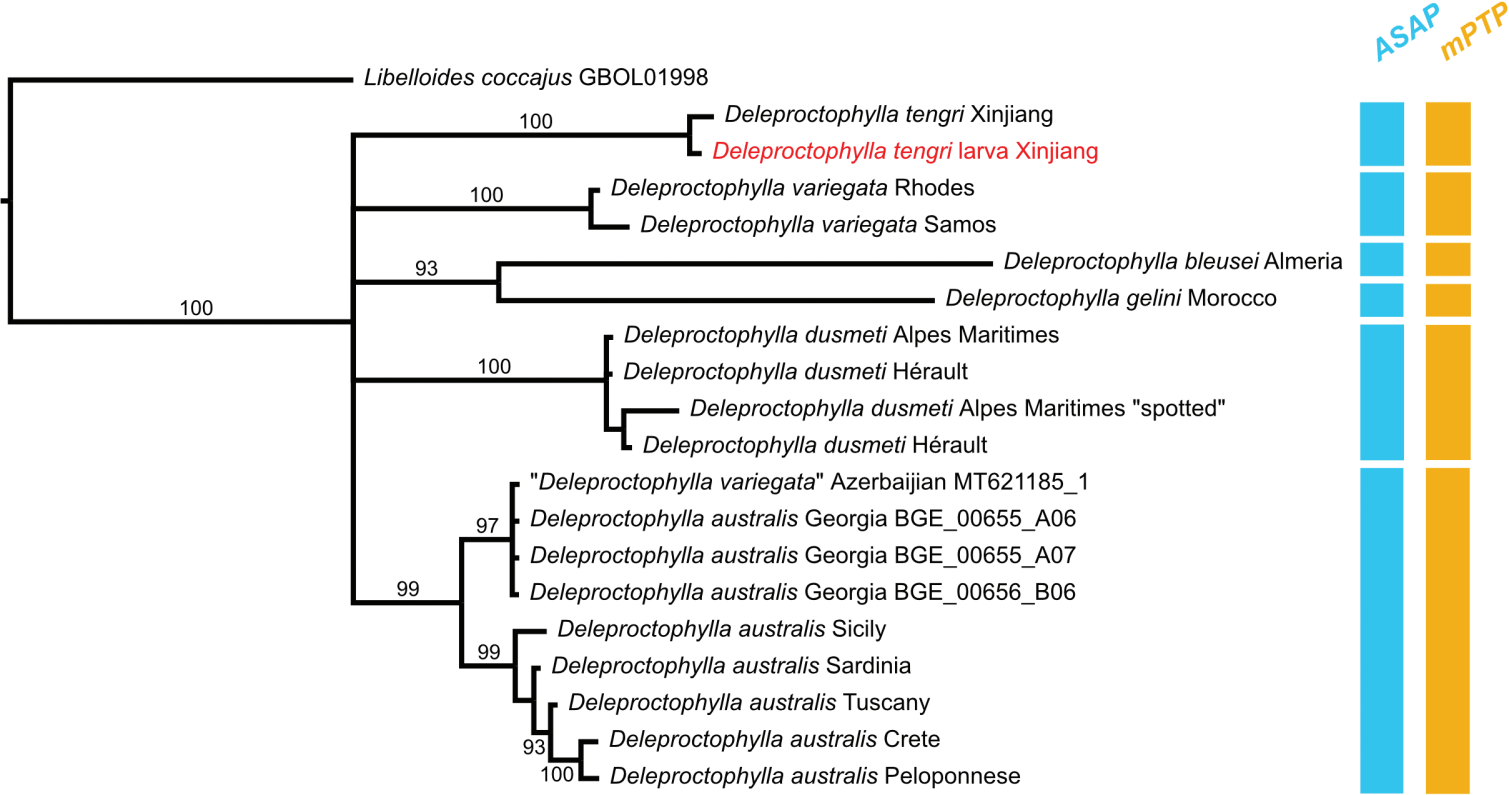
Bayesian phylogenetic tree of COI sequences of the analyzed specimens of *Deleproctophylla*. Bayesian posterior probabilities > 90 are shown above branches. Vertical bars indicate species delimitation analyses results obtained with ASAP and mPTP.

The BI analysis of the COI sequences (Table 1) strongly supported the monophyly of the six analyzed species of *Deleproctophylla* (Bayesian Posterior Probabilities: ≥ 99) (Fig. 1). The results of both species delimitation analyses based on the COI mitochondrial marker were congruent, identifying the same species across the sampled specimens. The best ASAP partition (ASAP score: 1.00, P-value: 2.994012e-01, W: 1) and the mPTP analysis consistently recovered six species within the sample (Fig. 1).

The phylogenetic analysis strongly supported that the examined larva collected in Xinjiang belongs to *D. tengri* sp. nov. (Fig. 1). Moreover, both ASAP and mPTP delimited *D. tengri* as a well distinct taxon.

Both methods confirmed that the population of *D. dusmeti* with atypical forewing markings belong to this species. Similarly, the analyses supported the monophyly of all examined *D. australis* specimens, including those collected from Mediterranean islands and the Caucasus. Notably, both methods identified a GenBank sequence from Azerbaijan, originally assigned to *D. variegata*, as deeply nested within *D. australis*, suggesting a misidentification. However, the Bayesian analysis recovered two well distinct sister clades within *D. australis*, one including all the European specimens and the other the Asian specimens.

The species delimitation analyses also supported the distinctiveness of the two species for which only singletons were available, namely *D. bleusei* and *D. variegata*.

### Taxonomy

#### Family Myrmeleontidae Latreille, 1807

**Subfamily Ascalaphinae Lefèbvre, 1842**

**Tribe Ascalaphini Lefèbvre, 1842**

##### Deleproctophylla

Taxon classificationAnimaliaNeuropteraMyrmeleontidae

Lefèbvre, 1842

BEA9AA02-FEDA-5955-B349-5403B133D1E3

Figs 2, 3, 4, 5, 6, 7, 8, 9, 10, 11, 12, 13, 14, 15, 16, 17, 18, 19, 20, 21, 22, 23, 24

Deleproctophylla Lefèbvre, 1842: 6 (emended to “Theleproctophylla” by Rambur, 1842: 351). Type species: Ascalaphus australis Fabricius, 1893, by subsequent designation by van der Weele (1908).

###### Diagnosis.

**Adult**. Head breadth at widest point, including compound eyes, slightly larger to width of mesothorax at wing base. Compound eye with deep median furrow, lateral margins slightly indented at furrow. Antenna not reaching pterostigma; club pyriform, wide. Forewing elongate; hindwing broadest at RP fork. Forewing membrane largely hyaline, with brown markings in a few species; hindwing membrane largely hyaline with brown markings or infuscations in apical half. Abdomen without pleuritocavae.

***Male genitalia***. Ectoproct with prominent “pincer-like” ventral projection, with bifurcating cylindrical branch in distal half, apex bent downward and inward; branch and apex with robust, spiniform setae. Tergite 9 sub-rectangular in lateral view, with prominent ventral thumb-shaped projection. Sternite 9 with caudal paired lateral lobes and a median, prominent sub-rectangular lobe; setae on median lobe spine-like. Complex of gx9+11 proportionally small in comparison to tip of abdomen; gx11 arch-shaped, projecting caudally and slightly curved downward in lateral view; gp11 forming a furrow, with short setae; gx9 widely set apart, with a dorso-apical, upward fold, resembling a smooth hook in lateral view.

***Female genitalia***. Ectoproct elongate, with short ventral projection. Tergite 9 subrectangular. Gx9 wider than long. Tergite 8 subrectangular. Gx8 distinct, not fused and widely set apart, wider than long, slightly prominent. Gp8 caudally prominent, appearing subtriangular in lateral view (except for *D. gelini*).

**Larva**. Head capsule not dilated posteriorly; ocular tubercle cylindrical with small apical protuberance; mandible with three teeth, median tooth largest and positioned closer to apical than to basal tooth; meso- and metathorax each with two pairs of sub-equal cylindrical scolus-like processus; anterior abdominal spiracle lateral; dorsal abdominal series of spiracles composed of eight cylindrical scolus-like processus, ventral series composed of two anterior scolus-like processus and six tubercle-like processus; sternite 8 with short odontoid processus; sternite 9 with short rastra with four digging setae; body dolichasters elongated, stick-shaped.

###### Comparative notes.

Potential synapomorphies of *Deleproctophylla* include: i) male ectoproct with caudal projection and median bifurcating branch, ii) male gx9 widely separated from each other and from gp11, with apical fold; iii) female ectoproct with caudal projection. *Deleproctophylla* is one of the few genera of Ascalaphini characterized by prominent male ectoproct with median branch, such as *Bubopsis* McLachlan, 1898, and *Phalascusa* Kolbe, 1897 (Tjeder 1992). However, in *Deleproctophylla* species, the ectoproct branch develops inward or dorsally (i.e., *D. gelini*), while in the sympatric species of *Bubopsis*, the branch extends basally and ventrally. These traits, combined with the characteristic pigmentation pattern of *Deleproctophylla*, allow easy identification from other ascalaphid genera with elongated ectoproct. In addition, to the obvious coloration differences, *Deleproctophylla* is immediately set apart from *Libelloides* in the shape of the male ectoproct, which is proportionally shorter, simpler in shape and lacking branch in the latter. However, these two genera share a similar conformation of the gx9+11 complex, especially in the shape of gx9. The larvae of these two genera are also very similar in body shape, tooth arrangement, and the position, development, and shape of thoracic and abdominal series of scolus-like processus. The larvae of *Deleproctophylla* can be distinguished by those of *Libelloides* in the head capsule, which is not dilated posteriorly and the dolichasters covering the body, which are mostly stick-shaped and do not expand apically into a wide rosette (Badano and Pantaleoni 2014).

###### Remarks.

**Figure 2.**
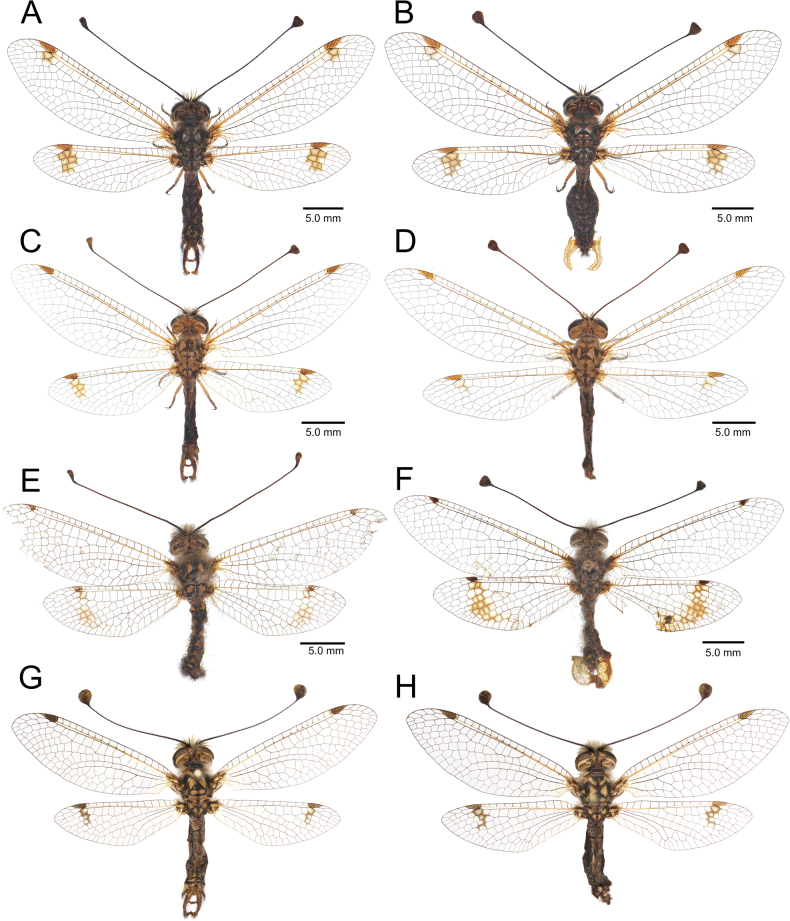
Habitus of *Deleproctophylla* spp. **A, C, E, G**. Male; **B, D, F, H**. Female; **A, B**. *D. australis* (Fabricius, 1787), Grosseto (Italy); **C, D**. *D. bleusei* Kimmins, 1949, Fes (Morocco); **E, F**. *D. dandizenor* Badano, Zheng, U. Aspöck & Dobosz, sp. nov., male holotype and female paratype, Kunar (Afghanistan); **G, H**. *D. dusmeti* (Navás, 1914), Saint-Paul-et-Valmalle (France).

**Figure 3.**
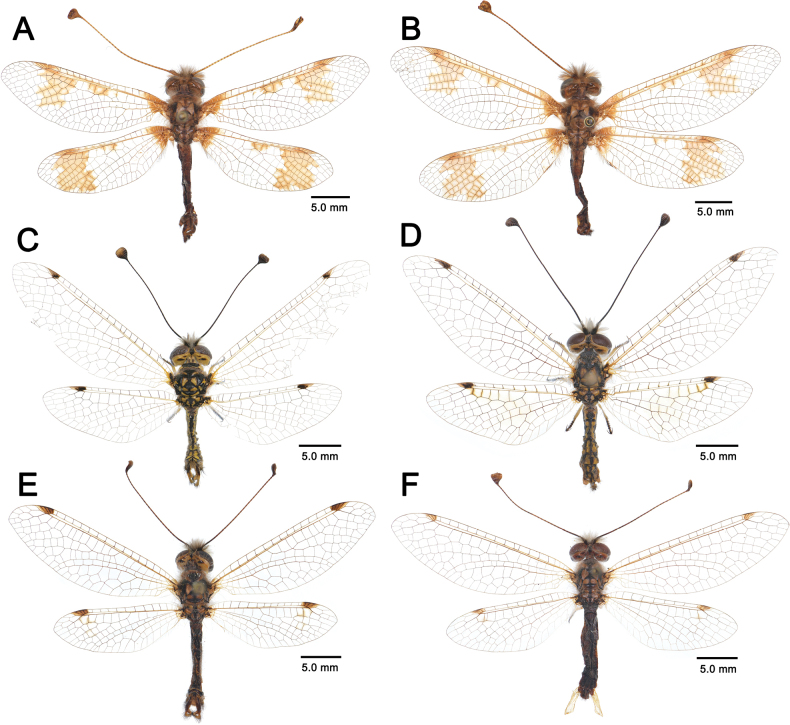
Habitus of *Deleproctophylla* spp. **A, C, E**. Male; **B, D, F**. Female; **A, B**. *D. gelini* (Navás, 1919), Guigou (Morocco); **C, D**. *D. tengri* Zheng, Badano, H. Aspöck & Liu, sp. nov., Xinjiang (China); **E, F**. *D. variegata* (Klug, 1834), Cyprus.

One of the most unusual features of *Deleproctophylla* is the presence of the so-called phylla, large, paired, membranous, leaf-shaped structures protruding from the female abdomen (Figs 2F, 3F) (H. Aspöck et al. 1980; Tjeder 1992). The genus name derives from this trait. The phylla consist of the male spermatophore, which remains attached to the female genital opening after mating but is shed when she lays eggs. The evolutionary significance of this prominent structure remains unclear, but it may serve to reduce competition between males by preventing remating with an already fertilized female. In a few other ascalaphid genera, such as *Bubopsis* McLachlan, females also retain the spermatophore after mating, but in none of them are these structures as large and striking as in *Deleproctophylla*.

### Key to the species of *Deleproctophylla* (adults)

**Table:** 

1	Vertex nearly as long as wide (Fig. 15C); forewing with large brown band extending for the whole RP area, hindwing with large marking extending for whole wing width and covering distal half of wing (Figs 3A, 3B, 15A); male ectoproct robust, strongly bent inward (Fig. 16A–C); female gp8 without prominent caudal margin (Fig. 16D, E)	***D. gelini***
–	Vertex longer than wide (Fig. 6C); forewing unmarked or with a small marking below pterostigma, hindwing with marking not covering distal half of wing, usually a small marking below pterostigma or narrow band (Figs 2, 3C–F, 4); male ectoproct not bent inward (Fig. 6A–C); female gp8 with prominent caudal margin (Fig. 7D, E)	**2**
2	Hindwing with indistinct marking, as a brown shade bordering crossveins below pterostigma, not extending along RP (Fig. 22A); male ectoproct with stout branch in apical two thirds of length and narrowing after branch bifurcation (Fig. 23A, B)	***D. variegata***
–	Hindwing with distinct marking, or as shade extending along RP (Fig. 2C, D); male ectoproct with stout branch in apical two thirds of length and not narrowing after branch bifurcation (Fig. 7A–C)	**3**
3	Metafemur without brown marking (Fig. 9D)	**4**
–	Metafemur with brown marking (Fig. 6D)	**5**
4	Forewing with round apex (Figs 2C, 2D, 4E, 4F, 9A); Hindwing marking distinct (Figs 2C, 2D, 4E, 4F, 9A); male ectoproct with long median branch after mid-length, covered with short and curved setae only on tip of branch and ectoproct (Fig. 10A, B)	***D. bleusei***
–	Forewing with acute apex (Figs 3C, 3D, 17A); hindwing marking generally as shade along crossveins (Figs 3C, 3D, 17A), extending on RP; male ectoproct median branch basally broad, gradually bifurcating, subtriangular in shape (Fig. 18A–C)	***D. tengri* sp. nov**.
5	Hindwing with marking extending as band for whole breadth of wing (Figs 2E, 2F, 11A); male ectoproct with wide median branch, densely covered with long robust setae on whole branch and apex of ectoproct (Fig. 12A–C)	***D. dandizenor* sp. nov**.
–	Hindwing with marking not extending beyond radial field (Fig. 4A–D, G, H); male ectoproct with narrow median branch, with curved short, covered with stout setae only at tip of branch and apex of ectoproct (Fig. 7A)	**6**
6	Hindwing elongate, hind margin not pronounced (Fig. 6A); male ectoproct with median branch bifurcating after mid-length (Fig. 7A, B); western populations usually with marked wings (Fig. 4A, 4C), eastern population with unmarked wings (Fig. 4B, 4D)	***D. australis***
–	Hindwing subtriangular, hind margin angled (Fig. 13A); male ectoproct with median branch bifurcating at mid-length (Fig. 14A, B); forewing without markings (Figs 2G, 2H, 4G), exceptionally marked in some populations (Fig. 4H)	***D. dusmeti***

### Key to the known larvae of *Deleproctophylla*

**Table:** 

1	Dorsal side of head capsule dark brown with paler markings, ventral side dark brown with three parallel longitudinal pale stripes (Fig. 5A–D)	***D. australis* and *D. dusmeti* (not differentiable)**
–	Dorsal side of head capsule pale brown mottled dark brown, ventral side uniformly pale brown (Fig. 20A, B)	***D. tengri* sp. nov**.

#### Deleproctophylla australis

Taxon classificationAnimaliaNeuropteraMyrmeleontidae

(Fabricius, 1787)

EE4BE285-C0D1-5529-BB4B-C86382DC91A5

**Figure 4.**
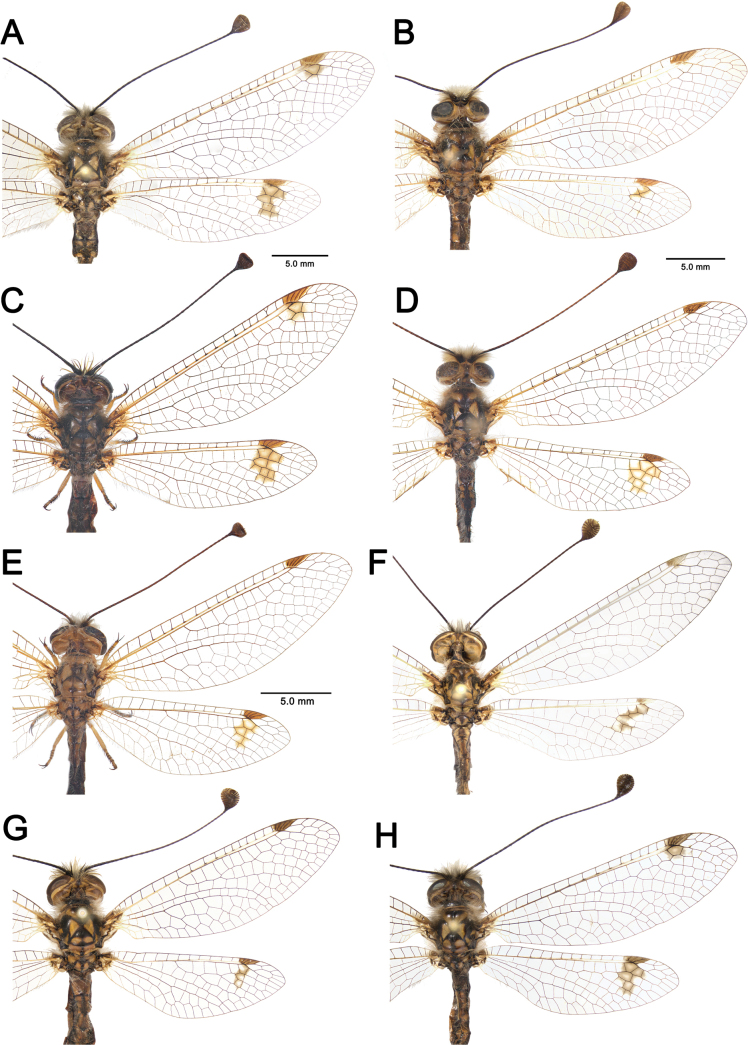
Variation of wing patterns in *Deleproctophylla* spp. **A**. *D. australis* (Fabricius, 1787), Crete (Greece); **B**. *D. australis*, Alghero, Sardinia (Italy); **C**. *D. australis*, Grosseto (Italy); **D**. *D. australis*, Balikesir (Turkey); **E**. *D. bleusei* Kimmins, 1949, Fes (Morocco); **F**. *D. bleusei*, Cabo de Gata (Spain); **G**. *D. dusmeti* (Navás, 1914), Saint-Paul-et-Valmalle (France); **H**. *D. dusmeti*, Villeneuve-Loubet (France).

**Figure 5.**
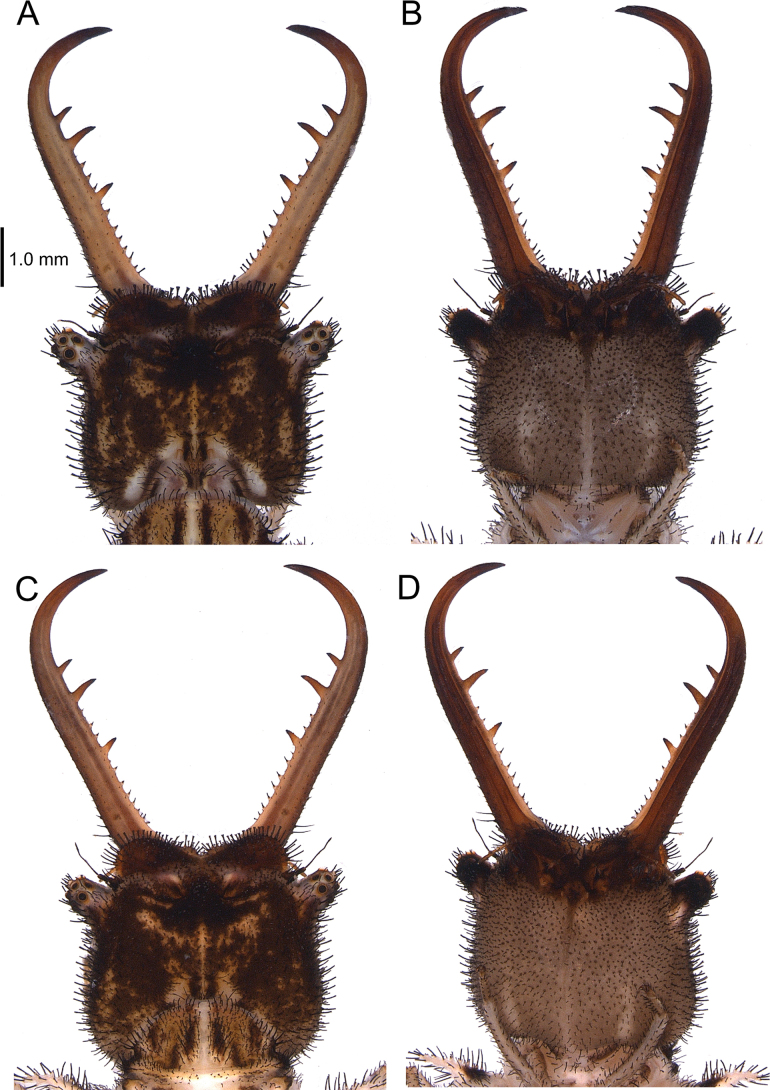
Head capsule of the larvae of *Deleproctophylla* spp. **A, B**. *D. australis* (Fabricius, 1787); **A**. Head, dorsal view; **B**. Head, ventral view; **C, D**. *D. dusmeti* (Navás, 1914); **C**. Head, dorsal view; **D**. Head, ventral view.

**Figure 6.**
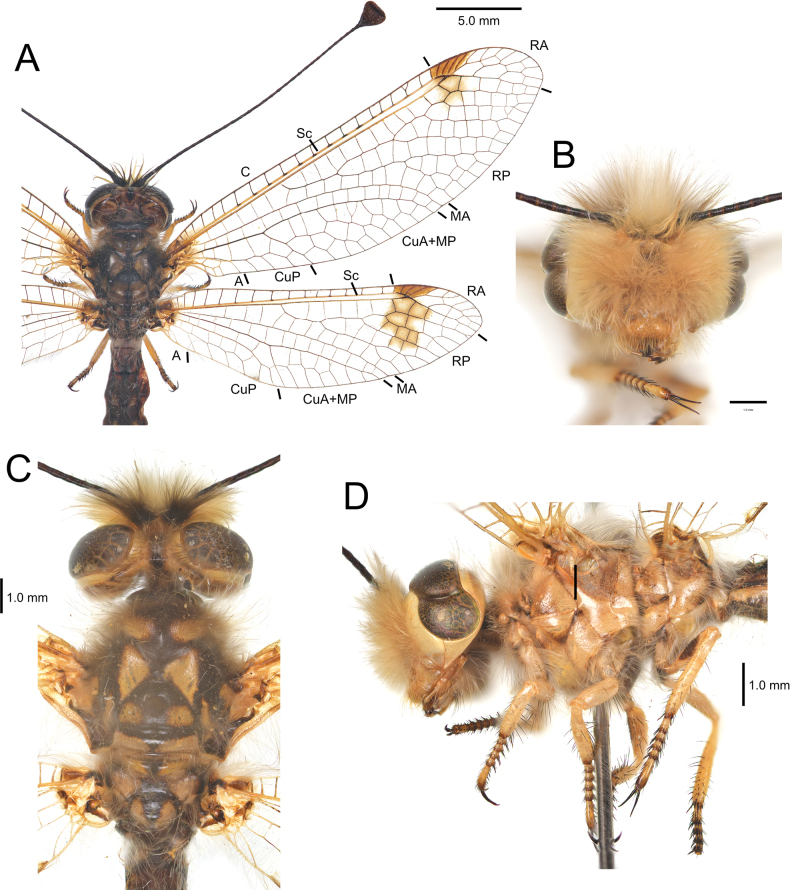
*Deleproctophylla australis* (Fabricius, 1787), adult. **A**. Habitus, Nomadelfia (Italy); **B**. Head, frontal view, Balikesir (Turkey); **C**. Head and thorax, dorsal view, Balikesir (Turkey); **D**. Head and thorax, lateral view. Abbreviations: C costa Sc subcosta RA radius anterior RP radius posterior MA media anterior MP media posterior CuA cubitus anterior CuP cubitus posterior A anal veins.

**Figure 7.**
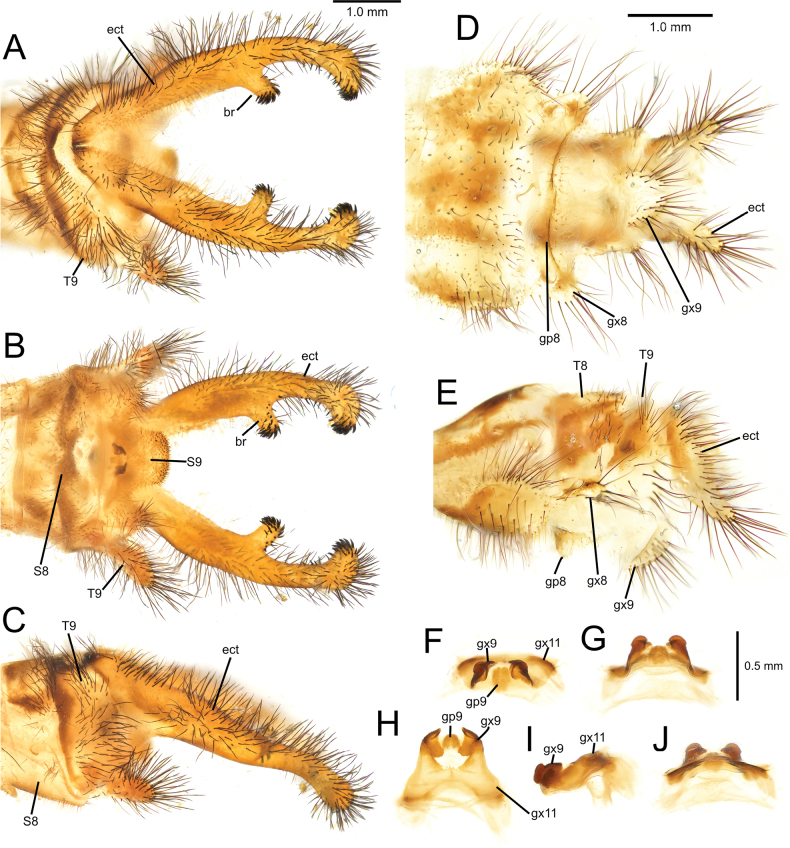
*Deleproctophylla australis* (Fabricius, 1787), adult, Nomadelfia (Italy). Male terminalia: **A**. Dorsal view; **B**. Ventral view; **C**. Lateral view. Female terminalia: **D**. Ventral view; **E**. Lateral view. Male genitalia: **F**. Ventral view; **G**. Anteroventral view; **H**. Caudal view; **I**. Lateral view; **J**. Dorsal view. Abbreviations: ect ectoproct br branch of ectoproct T8 tergite 8 S8 sternite 8 gx8 gonocoxite 8 gp8 gonapophysis 8 T9 tergite 9 S9 sternite 9 gx9 gonocoxite 9 gp9 gonoapophysis 9 gx11 gonocoxite 11.

**Figure 8.**
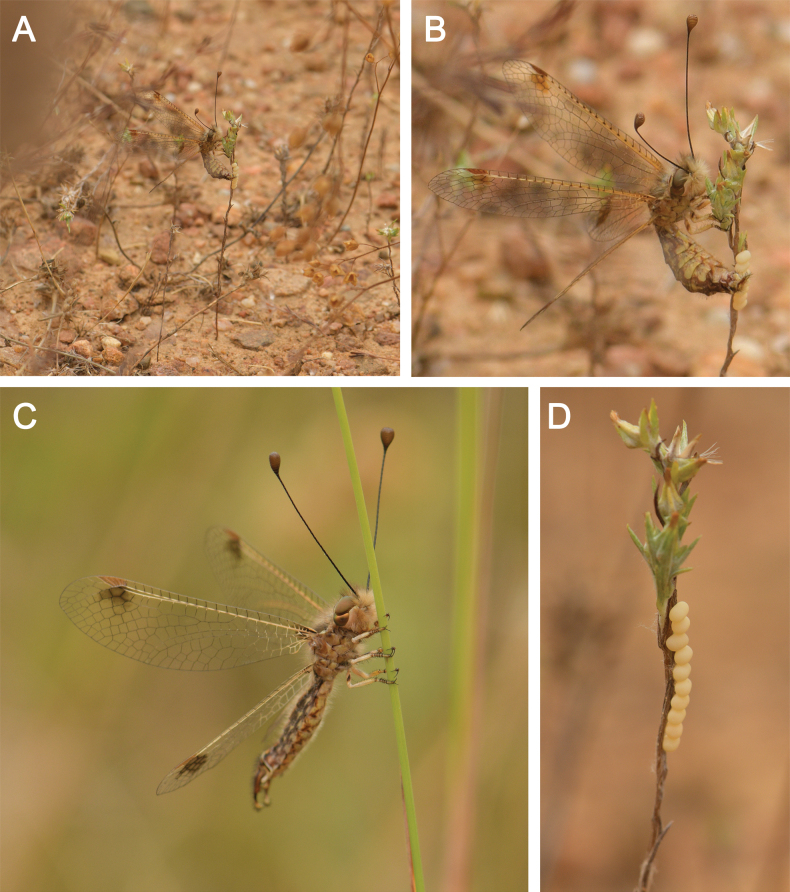
*Deleproctophylla australis* (Fabricius, 1787), live specimens, Nomadelfia (Italy). **A, B**. Female adult, laying eggs; **C**. Male adult; **D**. Eggs on a branch. Photos by YZ.

Figs 2A, 2B, 4A–D, 5A, 5B, 6–8

Ascalaphus australis Fabricius, 1787: 250 (type locality: “Europa australiore” = southern Europe; holotype in NHMD). Gmelin 1788: 2645 (Myrmeleon). Villers 1789: 62 (Myrmeleon). Olivier 1790: 245 (Ascalaphus). Fabricius 1793: 96 (Ascalaphus). Turton 1800: 405 (Myrmeleon). Angelini 1827: 468 (Ascalaphus). Lefèbvre 1842: 6 (Deleproctophylla). Rambur 1842: 351 (Theleproctophylla). Schneider 1845: 341 (Theleproctophylla). Walker 1853: 416 (Ascalaphus). Hagen 1860: 53 (Theleproctophylla). Stein 1863: 420 (Ascalaphus). Hagen 1866: 402 (Deleproctophylla). Costa 1866: 14 (Theleproctophylla). McLachlan 1873: 260 (Theleproctophylla). Navás 1905: 547 (Theleproctophylla). Navás 1911: 92 (Theleproctophylla). Navás 1912a: 124 (Theleproctophylla). Navás 1912b: 34 (Theleproctophylla). Lacroix 1913: 100 (Theleproctophylla). Kimmins 1930: 188 (Theleproctophylla). Mosely 1932: 182 (Theleproctophylla). Táborský 1936: 164 (Deleproctophylla). Capra 1936: 213 (Theleproctophylla). Capra 1937: 58 (Theleproctophylla). Zimsen 1964: 612 (Ascalaphus). Zelený 1964: 326 (Theleproctophylla). Popov 1977: 276 (Deleproctophylla). H. Aspöck et al. 1980: 315 (Deleproctophylla). Devetak 1992: 111 (Deleproctophylla). Zakharenko and Krivokhatsky 1993: 73 (Deleproctophylla). Bernardi et al. 1995: 11 (Deleproctophylla). Devetak 1995: 194 (Deleproctophylla). Letardi and Pantaleoni 1996: 299 (Deleproctophylla). H. Aspöck and Hölzel 1996: 78 (Deleproctophylla). Pantaleoni and Letardi 1998: 40 (Deleproctophylla). H. Aspöck et al. 2001: 301 (Deleproctophylla). Whittington 2002: 377 (Deleproctophylla). Popov 2004: 231 (Deleproctophylla). Canbulat 2007: 36 (Deleproctophylla). Dobosz and Ábrahám 2007: 24 (Deleproctophylla). Devetak and Janžekovič 2012: 183 (Deleproctophylla). Colombo et al. 2012: 125 (Deleproctophylla). Badano and Pantaleoni 2014: 301 (larva, Deleproctophylla). Letardi 2016: 194 (Deleproctophylla). Aistleitner 2019: 123 (Deleproctophylla). Jones 2019: 3 (Deleproctophylla). Poggi 2024: 9 (Deleproctophylla).
Theleproctophylla australis var. *impar* Navás, 1923: 154 (type locality: Greece: Thessaloniki; holotype in MNHN but lost at present).

##### Examined material.

• Croatia: Dalmatien, Hvar, 17.VII.1962, R. Löberbauer, 1 ♀ (HUAC); • Croatia: Dalmatien-Split, Küste, 22.VI.1953, H. Hamann, 1 ♂ 1 ♀ (HUAC); • Croatia: Ribnica, Otavice 10 km N v. Titov Velea, 28.VII.1965, 1 ♂ 1 ♀ (RMNH); Croatia: • Rovinj, 15.V.1953, 1 ♂ (HUAC); • Croatia: Ninski Stanovi, 40 m, 14.07.2004, L. Ábrahám, 31 ♂♂ 34 ♀♀(SCM); • Croatia: Zadar, Benkovac, 11.07.2002, D. Szalóki, 3 ♀♀ (SCM); • Croatia: Dalmacija, Gor. Poličnik, 12 km E of Zadar, 20.06.2007, leg. Zs. Józan, 1 ♀ (SCM); • Croatia: Dalmacija, Primošten-Bilo, 09.06.2003, Nikola Rahmé 1 ♂ (SCM); • Croatia: Vinodol Obc., Crikvenica, Selce, 180 m, 01.07.2001, K. Székely 1 ♂ (SCM); • Croatia: Ninski Stanovi, 40 m, 14.07.2004, L. Ábrahám, 3 ♂♂ 3 ♀♀ (USMB); • France: Corse, Calvi, 13–27.VII.1971, Fam. V. Oorschot, 1 ♀ (RMNH); • France: Corse, Belgodère, 350 m, 14.VII.1971, Fam. V. Oorschot, 1 ♀ (RMNH); • France: Corse, Belvedere, VII.1973, H.M. Steiner, 3 ♂ 1 ♀ (HUAC); • France: Corsica, Valle de Restino, 02.06.2000, leg. L. Nádai, 1 ♀ (SCM); • France: Haute-Corse, Castifao, 25.06.2002, leg. J. Bard, 1 ♀ (SCM); • Greece: Ahaia, Michas, 1300 m, 38°00'34"N, 21°53'00"E, 7.VII.2008, Dils J. and Faes J., 1 ♀ (RMNH); • Greece: Chalcide, Tristinika, 20.VI.2015, 1♂ (MDC); • Greece: Cos, Tigaki, 3–10.VII.1978, M. C. and G. Kruseman, 2 ♀ (RMNH); • Greece: Georgioupolis, 35°21'N, 24°16'E, H. u. L. Hölzel, 24 ♂ 19 ♀ (NHMW); • Greece: Georgioupolis, 35°21'N, 24°16'E, H. u. L. Hölzel, 1 ♂ 1 ♀ (HUAC); • Greece: Elis, Andritsaina, 1000 m, 29.VII.1978, L. Willemse and J. Tilmans, 1 ♂ (RMNH); • Greece: Ioannina, Mesovouni, 650 m, 39°56'18.8"N, 20°38'12.4"E, 28.VII.2006, T., W. and J. Garrevoet, 2 ♀ (RMNH); • Greece: Lesbos, Sigri, 6–8.VI.1988, J. P. Duffels, 1 ♂ 1 ♀ (RMNH); • Greece: Loutraki-Perachora, 38°N, 23°E, 30.V.1969, Rausch, 1 ♀ (HUAC); • Greece: Pierias, Litochoro, 5.VII.1976, F. and L. Willemse and J. Tilmans, 1 ♀ (RMNH); • Greece, Creta Nord, Hersonissos, 35°18'36.3"N, 25°23'53.9"E, 15.V.2024, J. Vetrovec, 1 ♂ (DB); • Greece: Creta, Lasithi, Xerokampos, 2.VI.1972, M. C. and G. Kruseman, 1 ♂ 2 ♀ (RMNH); • Greece: Kardaras, 800 m, 37°39'22.9"N, 22°18'34.2"E, 7.VII.2008, Dils J. and Faes J., 1 ♂ (RMNH); • Greece: Tripolis-Davia 3–8 km NW of Tripolis, 800 m, 13.VII.1975, J. P. Duffels, 1 ♀ (RMNH); • Greece: Leptokaria, 28.06.1992, Balásházy, 1 ♀ (SCM); • Greece: Sithonia, Sarti, 0–50 m, 02–09.07.1997, D. Szalóki, 1 ♂ 4 ♀♀ (SCM); • Greece: Sithonia, Sarti, 0–50 m, 02–09.07.1997, D. Szalóki, 1 ♀ (USMB); • Greece: Argolída, SE of Néa Epídavros, 37°40'N, 23°09'E, 120 m, 09.06.2007, R. Królik, 1 ♂ (USMB); • Greece: Corfu, Acharavi [CK90], xerothermic meadow, 06.07.2011, W. Żyła, 3 ♂♂ (USMB); • Greece: Corfu, Acharavi [CK90], xerothermic meadow, 07.07.2011, W. Żyła, 1 ♂ (USMB); • Greece: Chalkidiki, Paliouri env., 39°56'31"N, 23°39'29"E, SE of Kassandria, 06–11.06.2013, M. Šnižek, 2 ♂♂ (USMB); • Greece: Chalkidiki, Paliouri env., 39°56'31"N, 23°39'29"E, SE of Kassandria, 12–14.06.2013, M. Šnižek, 3 ♂♂ (USMB); • Greece: Thessaly, Kokkino Nero, 39°49'N, 22°47'E, environs, 17–29.05.2015, A. Skiba, 1 ♂ (USMB); • Greece: Chalkidiki, E of Paliouri, 39°56'N, 23°41'E, Kassandria, 11.06.2022, M. Šnižek, 1 ♂ (USMB); • Greece: Peloponnese, Loutra Killinis, 37°51'12"N, 21°6'37"E, dunes and grass, 19.06.2023, W. Żyła, 1 ♀ (USMB); • Greece: Peloponnese, Messenia, SE of Kalamia, 37°52'13.5"N, 21°07'08.7"E, olive garden, 13.06.2024, W. Żyła, 2 ♀♀ (USMB); • Greece: Peloponnese, Messenia, SE of Kalamia, 37°52'13.5"N, 21°07'08.7"E, olive garden, 14.06.2024, W. Żyła, 1 ♂ 1 ♀ (in absolute alcohol) (USMB); • Italy: Basilicata, 10 km uoru, Potenza, 700 m, 40°35'N, 15°45'E, 1 ♂ (HUAC); • Italy: Calabria, Crotone, Catanzaro, 30–100 m, 38°55'N, 16°55'E, 4.VIII, 1 ♂ (HUAC); • Italy: Prov. Messina, Lipari, Isole, Stromboli, San Vincenzo, 17.VII.1995, Alain Olivier, 1 ♀ (RMNH); • Italy: Tuscany, Grosseto, Nomadelfia (GR), 42°50'23"N, 11°08'50"E, 29.VI.2024, Davide Badano and Yuchen Zheng, 2 ♂ 2 ♀ (ZCAU); • same locality • 1 ♂ 3 ♀ (DB); • Italy: Tuscany, Grosseto, Roselle (GR), 42°49'34"N, 11°10'05"E, 29.VI.2024, Davide Badano and Yuchen Zheng, 1 ♂ 2 ♀ (ZCAU); • same locality data • 1 ♀ (DB); • Italy: Tuscany, Grosseto, Parco Nazionale della Maremma, Marina di Alberese (GR), 42°39'51"N, 11°02'25"E 20.VI.2025, Davide Badano leg., 1 ♂ 2 ♀ (DB); • Italy: Lazio, Roma (RM), Via Appia Antica, 11.VII.1937, L. Barbera, 1 ♀ (MSNG); • Italy: Nomadelfia (GR), 42°50'23.2"N, 11°08'51.4"E, 30.VI.2024, D. Badano and Y. Zheng, (DB); • Italy: Puglia, Bari, Masseria Pellicciari, 450 m, 9.VII.2000, Fontana P., Kleukers R. and Cogo A., 1 ♀ (RMNH); • Italy: Puglia, Castel del Monte, 450 m, 41°05'20.9"N, 16°16'16.3"E, 2.VII.2001, T. and W. Garrevoet, 1 ♀ (RMNH); • Italy: Prov. Salerno, Pioppi, Cost Cilentana, 14.VII.1971, Holzinger, 1 ♀ (HUAC); • Italy: Sardegna, Marrubiu (OR), Sant’Anna, stazione e dintorni, 1.VI.2003, C. Meloni, 1 same locality, ♂ (MSNG); • same locality, 2.VI.2006, 1 ♀ (MSNG); • same locality, 26.VI.2009, C. Meloni, 3 ♂ 12 ♀ (MSNG); • Italy: Sardegna, Alghero (SS), Lu Cantar, 40°32'25.3"N, 8°19'29.7"E, 12.VII.2012, D. Badano, 3 ♂ (DB); • Italy: Sardegna, Alghero (SS), Monte Doglia, 40°36'20.1"N, 8°15'06.0"E, 7.VII.2012, D. Badano, 3 ♀ (DB); • Italy: Sardegna, Monti (OT), 2.VII.2011, D. Badano, 2 ♀ (DB); • Italy: Sicilia, Sud di Castrofilippo (AG), m 450, 4.VI.2015, E. Gallo, 1 ♀ (MSNG); • Italy: Sicilia, Caltanissetta (CL), strada Enna, bivio Gela, 8.VII.1954, Adalberto Parvis, 2 ♂ 3 ♀ (MSNG); • Italy: Sicilia, Peloritani, 10 km SW Barcellona, 100 m, 38.06°N, 15.07°E, 25–26.1974, 2 ♂ 2 ♀ (HUAC); • Italy: Sardinia, **Siniscola, Su Tilio**, 27.06.2004, V. Feik, 1 ♀ (NMPC); • Italy: Sardinia, “Sardinien, Mti Gennargentu Mte, Spada”, 1250 m, 17–21.06.1968, Ströhle, 2 ♂♂ (SCM); • North Macedonia: Demir Kapija, Barovo, 27.VII.1965, 1 ♀ (RMNH); • North Macedonia: Štip, 23.VII.1965, 1 ♂ (RMNH); • Turkey: Aydin, Kusadasi, 11.VII.1980, H. v. Oorschot, 1 ♀ (RMNH); • Turkey: Balıkesir, nahe Bezirci (zw. Edincik u. Bugdayli), 150 m, 40.15°N, 27.47°E, 22.VII.1978, H.and U. Aspöck, H and R Rausch, P. Ressl, 3 ♂ 4 ♀ (HUAC); • Turkey: Edirne, 08–13.06.[19]47, 1 ♂, Exp. N. Mus. ČRS (NMPC).

##### Diagnostic characters.

Metafemur with brown marking; forewing with brown marking below pterostigma (often absent in eastern populations); hindwing elongate, posterior margin curved; not angled; hindwing with large marking below pterostigma; male ectoproct with branch in apical half of branch, after mid-length; setae on branch only at tip; setae on branch and tip of ectoproct stout and curved.

##### Redescription.

***Size*** (mm, based on 5 specimens). Head + thorax length 7.7–8.6; antenna length 15.1–2; fore wing length 21.7–22.9; fore wing width 6.4–7.4; hindwing length 17.1–18.7; hindwing width 5.0–5.7.

***Head***. Vertex yellowish brown; setae dense, long, pale yellow (Fig. 6C). Para-ocular band pale yellow. Frons yellowish brown; setae dense, long, pale yellow. Clypeus and labrum yellowish brown; setae long, pale (Fig. 6B). Mandible yellowish brown, transitioning to dark brown apically. Labium and palp yellowish brown, concolorous with mandible. Antenna with pedicel with dense pale and dark setae, flagellomeres brown, nodes slightly darker; club brown, anterior face paler (Fig. 6A).

***Thorax***. Cervical sclerite yellowish brown. Pronotum brown; setae long, pale yellow. Mesonotum brown with yellowish brown markings: an anterior rounded pair, a median triangular pair, and a posterior rounded pair; mesoscutellum anteriorly dark brown, posteriorly yellowish brown. Mesonotum with long pale yellow setae. Metanotum largely yellowish brown, medially dark brown; setae pale yellow (Fig. 6C). Pleurae yellowish brown; setae pale yellow (Fig. 6D).

***Legs***. Pro- and mesothoracic legs yellowish brown, tarsi slightly darker. Metathoracic leg yellowish brown, metafemur with dorsal dark brown marking. Coxae with thin pale yellow setae; femora and tibiae with thin pale yellow setae and ventral thicker blackish setae; tibial spurs narrow, blackish; tarsi with robust blackish setae (Fig. 6D).

***Wings***. Forewing long, apex rounded. Pterostigma with four to five forked and unforked brown veinlets; membrane brown. Presectoral area with ~6 crossveins. RP with five branches. MA gradually curving toward hind margin in distal portion. MP-CuA area with ~3 irregular rows of cells. Anal area hind margin straight; axillary lobe slightly pronounced. Forewing venation largely dark brown, paler toward wing base; Sc and R light brown; anal veins pale brown. Forewing membrane hyaline (in old specimens with faint brownish), distal section usually with a brown marking in radial area, just below pterostigma (absent in some populations) (Figs 2A, 2B, 4A–D, 6A). Hindwing elongated, broadest at RP fork; hind margin smoothly curved gradually narrowing toward rounded apex. Presectoral area with three crossveins. RP with four branches. MA largely straight, slightly curved toward hind margin distally. MP-CuA area with ~2 irregular rows of cells. Hindwing venation dark brown, paler near wing base. Hindwing membrane hyaline, distal section with a rather large brown marking extending from pterostigma across radial area until first branch of RP (Figs 2A, 2B, 4A–D, 6A).

***Abdomen***. Tergites pale brown, each with a caudal pair of yellowish markings. Sternites pale brown, paler than tergites. Pleural membrane brown with large paler markings.

***Male genitalia***. Ectoproct narrow, with branch in apical half, after mid-length. Setae of ectoproct long, dark brown; setae on branch limited to tip, dense, black, stout, curved, and spine-like; setae only on tip of ectoproct, black, stout, curved, and spine-like (Fig. 7A–C). Tergite 9 sub-rectangular in lateral view, with ventral projection; setae long and black, longer and denser on projection. Sternite 9 with three lobes, of which median largest; setae thin, spine-like on median lobe (Fig. 7B, C). Gx11 arch-shaped, slightly curved downward in lateral view; gx9 strongly sclerotized, with upward fold; gp9 lightly sclerotized forming a shallow median furrow, with short setae (Fig. 7F–J).

***Female genitalia***. Ectoproct elongated; setae long, black (Fig. 7D, E). Tergite 9 sub-rectangular. Gx9 wider than long ventrally; setae long. Tergite 8 subrectangular. Gx8 wider than long, with long setae. Gp8 prominent; setae thin (Fig. 7D, E).

**Larva**. See Badano and Pantaleoni (2014) (Fig. 5A, B).

##### Distribution.

Asia: Azerbaijan (see remarks for *D. variegata*), Georgia, Turkey (see remarks). Europe: Albania, Bulgaria, Croatia, France (Corsica), Greece, Italy (including Sardinia and Sicily), North Macedonia, Turkey (Fig. 25) (H. Aspöck et al. 1980; Devetak 1995; H. Aspöck et al. 2001; Popov 2004; Devetak and Janžekovič 2012; Kerimova et al. 2022; Japaridze et al. 2024; Oswald 2025).

##### Life history notes.

*Deleproctophylla australis* is typically associated with arid grasslands, meadows, and clearings (H. Aspöck et al. 1980; Devetak 1995; Popov 2004) (Fig. 8). *Deleproctophylla australis* is usually active at daylight, despite flights at dusk with hot weather. The female seems to prefer ovipositing on short grass stems (Fig. 8A, B), the eggs are placed in rows, as typical for the tribe (Fig. 8D).

##### Remarks.

*Deleproctophylla australis* is the type and first described species of *Deleproctophylla*. Among all congeners, *D. australis* is also the species with the widest distribution, ranging from the Tyrrhenian islands to western Asia (Fig. 25). However, this owlfly is usually localized and not particularly common (H. Aspöck et al. 1980, 2001).

The reliability of the forewing marks as a diagnostic character of *D. australis* is challenged by its absence in some specimens both due to individual variation and geographic differences (Fig. 4A–D). *Deleproctophylla australis* is distinguished from congeners, particularly the highly similar *D. dusmeti*, by a combination of wing and genital characters; *D. australis* is characterized by the elongated hindwing without a prominent posterior angle (present in *D. dusmeti*) and male ectoproct with branch arising after mid-length (at mid-length in *D. dusmeti*). Western populations of *D. australis* are typically characterized by the presence of a forewing spot below pterostigma, a trait often considered diagnostic in distinguishing this species from its European congeners (e.g., H. Aspöck et al. 1980) (Figs 2A, 2B, 4A–D, 6A). However, the frequency of this forewing marking diminishes eastward, with the populations of the Balkan Peninsula including spotted and unspotted specimens and those of Anatolia lacking the spot (Fig. 4D). These Asian unmarked specimens were assigned to a “*dusmeti*-like morph”, due to the shared absence of forewing marking and presence of spot on metafemur (H. Aspöck et al. 1980). The re-examination of specimens from these populations suggests that these enigmatic individuals from Anatolia belong to *D. australis*, as supported by wing shape and characters of male genitalia. Nevertheless, specimens of *D. australis* without spotted fore wings can be present in all populations, including Italy (Fig. 4B).

#### Deleproctophylla bleusei

Taxon classificationAnimaliaNeuropteraMyrmeleontidae

Kimmins, 1949

81ED67A9-402A-5FD4-A0B8-B49545A71696

**Figure 9.**
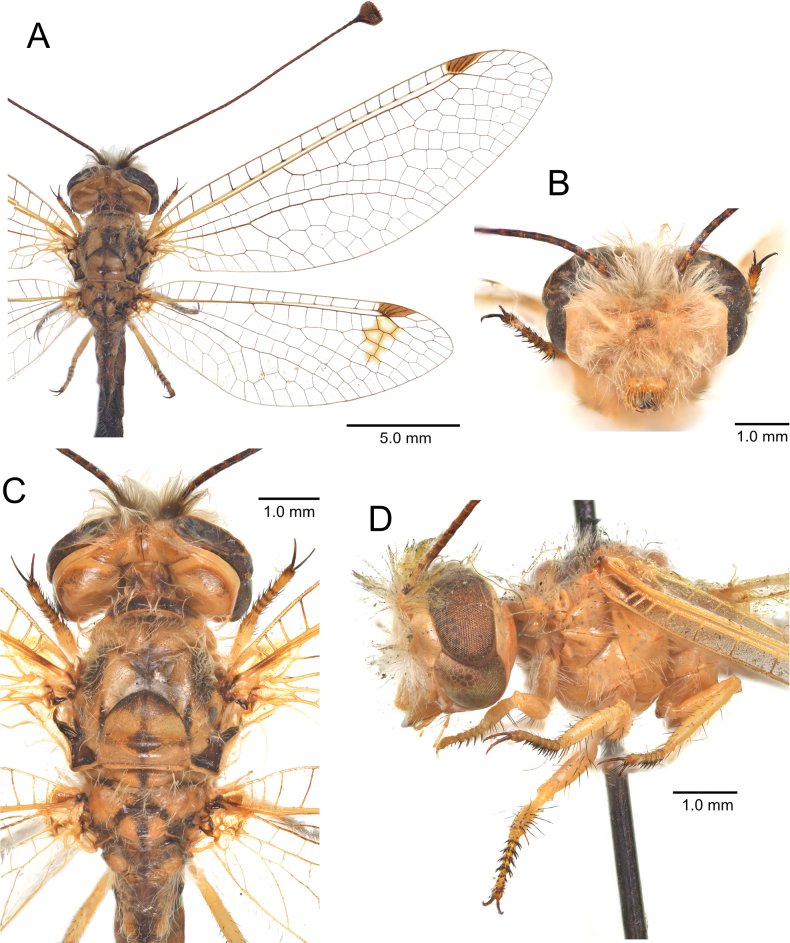
*Deleproctophylla bleusei* Kimmins, 1949, Fes (Morocco). **A**. Habitus; **B**. Head, frontal view; **C**. Head and thorax, dorsal view; **D**. Head and thorax, lateral view.

**Figure 10.**
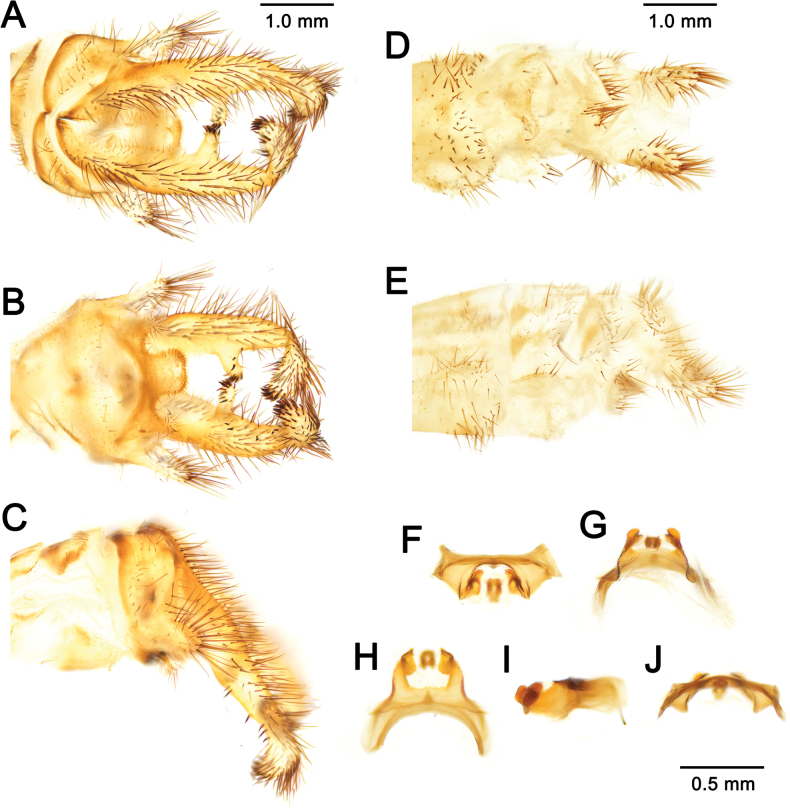
*Deleproctophylla bleusei* Kimmins, 1949, Fes (Morocco). Male terminalia: **A**. Dorsal view; **B**. Ventral view; **C**. Lateral view. Female terminalia: **D**. Ventral view; **E**. Lateral view. Male genitalia: **F**. Ventral view; **G**. Anteroventral view; **H**. Caudal view; **I**. Lateral view; **J**. Dorsal view.

Figs 2C, 2D, 4E, 4F, 9, 10, 24A, 24B

Deleproctophylla bleusei Kimmins, 1949: 28 (type locality: Algeria: Naama, Mécheria; holotype in NHMUK). H. Aspöck and Hölzel 1996: 78 (Deleproctophylla). H. Aspöck et al. 2001: 302 (Deleproctophylla). Güsten 2003: 133 (Deleproctophylla). Monserrat et al. 2014: 157 (Deleproctophylla). Monserrat 2015: 478 (Deleproctophylla). Michel and François 2019: 183 (Deleproctophylla). Monserrat et al. 2022: 81 (Deleproctophylla).

##### Material examined.

**Type specimen: *Holotype***. Images available at https://data.nhm.ac.uk/object/73295e3e-26cd-4bf4-8c10-0879722377ff.

##### Additional material.

• Morocco: Fes-el-Bali (60 km SE Ouezzane), 34.38°N, 5.16°W, 300 m, 8.VII.1977, 4 ♂, 6 ♀ (HUAC); • Spain: Alicante, Cox, VIII.1911, Andréu, 1 ♂ (MCNM); • Morocco: Middle Atlas, Parc National de Tazekka, env. of Barrage Bab Louta Lake, 34°0'5.90"N, 4°18'28.70"W, 590 m a.s.l., dry, ruderal meadow, 15.06.2022, 2 ♂, W. Szczepański (USMB); • some locality, date, 1 ♂, W. Szczepański (SCM); • Spain: Almería, Cabo de Gata, 30SWF67, 27.V.2010, F. Rodríguez and J.R. Correas, 1 ♂ (VM); • same locality 16.VI.2013, 2 ♂, 1 ♀ (VM); • same locality 6.VII.2013, 1 ♂, 2 ♀ (VM), 1 ♀ (DB); • Almería, Cabo de Gata, Salinas, Las Amoladeras, 30SWF67, 14.VI.2012, 1 ♀ (VM); • same locality, 6.VII.2013, 2 ♀ (VM); • same locality, 30.VII.2013, 1 ♀ 1 ♂ (VM); • Spain: Almería, San José, Playa de los Genoveses, 14.VI.2012, 3 ♂ 2 ♀ (VM); • Spain: Almería, San José, Playa de Mónsul, 10 m, 14.VI.2012, 1 ♀ (VM); • Spain: Murcia, Lorca, Purias, 30SXG25, 550 m, 12.VI.2010, José Carrillo, 1 ♀(VM); • same locality, 19.VI.2010, José Carrillo, 1 ♂ (VM).

##### Diagnostic characters.

Metafemur unmarked; forewing unmarked; hindwing posterior margin markedly curved, convex; hindwing with marking below pterostigma; male ectoproct with branch long, in apical half of branch, just after mid-length; setae on branch only at tip; setae on branch and tip of ectoproct stout and curved.

##### Redescription.

***Size*** (mm, based on 4 specimens) Head + thorax length 6.7–7.1; antenna length 11.6–14.5; fore wing length 15.7–22.4; fore wing width 6.5–6.7; hindwing length 19.2–22.7; hindwing width 4.2–5.5.

***Head***. Occiput yellowish brown; setae dense, long, pale yellow (Fig. 9C). Vertex yellowish brown; setae dense, long, pale yellow (Fig. 9B). Para-ocular band pale yellow. Frons yellowish brown; setae dense, long, pale yellow. Clypeus and labrum yellowish brown; setae long, pale. Mandible yellowish brown, darker apically. Labium and palp yellowish brown. Antenna with pedicel with dense pale and dark setae, flagellomeres brown, nodes slightly darker; club brown, paler anteriorly (Fig. 9A).

***Thorax***. Cervical sclerite yellowish brown. Pronotum brown; setae long, pale yellow. Mesonotum yellowish brown; internal margins of sclerites with darker brown shades; mesoscutellum yellowish brown with faint median brown stripe; setae long, pale yellow. Metanotum yellowish brown, with faint median marking; setae pale yellow (Fig. 9C). Pleurae yellowish brown; setae pale yellow (Fig. 9D).

***Legs***. Pro- and mesothoracic legs entirely yellowish brown. Metathoracic leg yellowish brown, unmarked. Setae of coxae pale yellow; setae of femora and tibiae thin, pale yellow, thicker blackish setae ventrally; tibial spurs narrow; setae of tarsi blackish (Fig. 9D).

***Wings***. Forewing long, apex rounded. Pterostigma with ~4 forked and unforked brown veinlets; membrane very pale brown, almost whitish in young (but not freshly emerged) individuals. Presectoral area with ~5 crossveins. RP with five branches. MA progressively curving toward hind margin in distal portion. MP-CuA area with ~3 irregular rows of cells. Axillary lobe slightly prominent. Forewing venation largely brown, paler toward wing base; Sc and R yellowish, very pale in young individuals; anal veins yellowish. Forewing membrane hyaline (in old specimens faint brownish), unmarked (Figs 2C, 2D, 4E, 4F, 9A). Hindwing broad, wider at RP fork; hind margin markedly convex, with a pronounced curve. Presectoral area with three crossveins. RP with four branches. MA largely straight, slightly curved toward hind margin distally. MP-CuA area with ~2 irregular rows of cells. Hindwing venation brown, paler near wing base; Sc yellowish. Hindwing membrane hyaline, distal section with a long narrow brown marking, sometimes stripe-like, extending from pterostigma to first branch of RP (Figs 2C, 2D, 4E, 4F, 9A).

***Abdomen***. Tergites pale brown, each with a caudal pair of yellowish markings. Sternites pale brown, paler than tergites. Pleural membrane pale brown.

***Male genitalia***. Ectoproct narrow, with long and thin branch bifurcating beyond mid-length, in apical half. Setae of ectoproct long, dark brown; setae on branch only at apex, dense, black, stout, curved, and spine-like; setae on tip of ectoproct black, stout, curved, and spine-like (Fig. 10A–C). Tergite 9 sub-rectangular in lateral view, with prominent ventral projection; setae long and black, longer and denser on projection. Sternite 9 with three lobes, of which median largest; setae thin, spine-like on median lobe (Fig. 10B, C). Gx11 arch-shaped, projecting caudally and curved downward laterally; gx9 sclerotized, dorsally folded; gp9 lightly sclerotized forming a shallow median furrow (Fig. 10F–J).

***Female genitalia***. Ectoproct elongated; setae long, black (Fig. 10D, E). Tergite 9 sub-rectangular. Gx9 wider than long; setae long. Tergite 8 subrectangular. Gx8 wider than long, with long setae. Gp8 projecting ventrally; setae thin (Fig. 10D, E).

##### Comparative notes.

*Deleproctophylla bleusei* has a long history of taxonomic confusion with *D. dusmeti*, to which it is sympatric in the Iberian Peninsula and closely resembles in wing pattern (Monserrat et al. 2014). However, *D. bleusei* can be distinguished by the unmarked metafemur (Fig. 9D) and characters of male ectoproct, such as long and thin branch arising after mid-length (Fig. 10A). The shape of the hindwing provides a reliable diagnostic trait: *D. bleusei* has smoothly curved posterior margin in *D. bleusei*, whereas *D. dusmeti* is characterized by a distinctly triangular shape. Additionally, young (but not newly hatched) specimens of *D. bleusei* are characterized by a pale C, Sc and R veins, as well as an almost whitish pterostigma. This distinctive venation coloration is also evident in the holotype.

##### Distribution.

Africa: Algeria, Morocco, Tunisia. Europe: Spain (H. Aspöck et al. 2001; Monserrat et al. 2014; Oswald 2025) (Fig. 25).

#### Deleproctophylla dandizenor

Taxon classificationAnimaliaNeuropteraMyrmeleontidae

Badano, Zheng, U. Aspöck & Dobosz sp. nov.

40445BE1-F3D7-56FD-8718-58C579950214

https://zoobank.org/D2497E1D-FABE-4B44-9F61-93B1E542BC5F

Figs 2E, 2F, 11, 12

##### Type material.

***Holotype*** • Afghanistan: Pr. Kunar, Nuristan ob. LindaiSin-Tal vic. Barg e Matal, Dandizenor Mts., 2600 m, 13.VII.1970, Naumann 1 ♂ (NHMW); paratype, same information as holotype, 1 ♀ (NHMW). ***Paratypes*** • Afghanistan: Province Bamiyan, Yakawlang District, SW Kotak village, ca 2800 m, 03.07.2016, I. Pljushtch, 1 ♂ (SCM); • Afghanistan: Province Bamiyan, Bande-Amir National Park, 2950 m, 26.06.2016, I. Pljushtch, 1 ♂ (USMB); • Pakistan: Hindukush Mts., Booni, 2000 m, 11.07.1994, B. Herczig, Gy. M. László and G. Ronkay, 1 ♂ (SCM).

##### Diagnosis.

Metafemur with large brown marking; pterostigma of both wings dark brown; wing venation rather dense, with many crossveins; forewing hyaline; hindwing broad; hindwing with large band-shaped marking from pterostigma to posterior margin of wing; male ectoproct robust, with branch in apical two-thirds of branch; ectoproct, including branch and apex densely covered with setae; setae on branch and apex spine-like. Male tergite 9 with caudal margin with long hair-like setae, ventral projection stout, hairy. Female ectoproct with short ventral projection. Female gx9 and gp8 relatively prominent.

##### Description.

***Size*** (mm, based on 2 specimens) Head + thorax length 8.1–8.2; antenna length 15.4–15.5; fore wing length 20.3–21.9; fore wing width 6.5–6.8; hindwing length 15.5–17.7; hindwing width 5.4–5.5.

**Figure 11.**
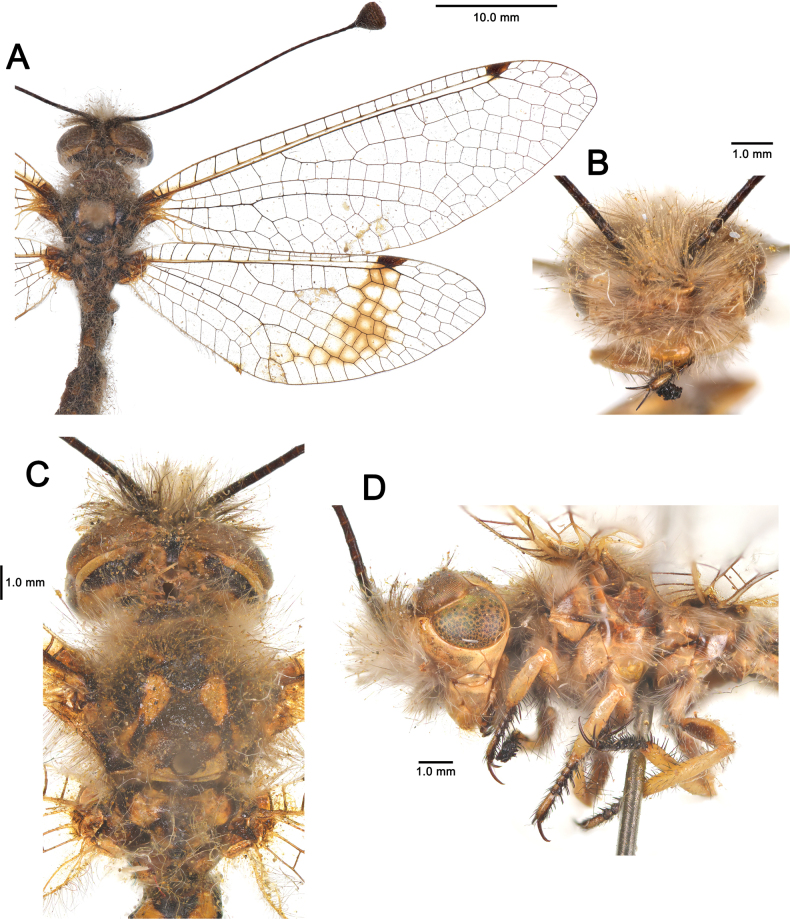
*Deleproctophylla dandizenor* Badano, Zheng, U. Aspöck & Dobosz, sp. nov., Kunar (Afghanistan). **A**. Habitus; **B**. Head, frontal view; **C**. Head and thorax, dorsal view; **D**. Head and thorax, lateral view.

***Head***. Vertex yellowish brown; setae dense, long, pale yellow (Fig. 11C). Para-ocular band yellowish brown, darker anteriorly. Frons yellowish brown; setae dense, long, pale yellow. Clypeus and labrum yellowish brown; setae long, pale (Fig. 11B). Mandible yellowish brown, transitioning to brown apically. Labium and palp yellowish brown, concolorous with mandible. Antenna with pedicel with pale and dark setae, flagellomeres brown; club brown (Fig. 11A).

***Thorax***. Cervical sclerite yellowish brown. Pronotum brown; setae long, pale yellow. Mesonotum brown with yellowish brown markings: an anterior small, rounded pair, a median subtriangular pair, and a posterior rounded pair; mesoscutellum dark brown, caudal margin yellowish brown. Mesonotum with long pale-yellow setae. Metanotum brown, medially dark brown; setae pale yellow (Fig. 11C). Pleurae yellowish brown; setae pale yellow (Fig. 11D).

***Legs***. Pro- and mesothoracic legs yellowish brown, tarsi dark brown. Metathoracic leg yellowish brown, metafemur with large dark brown marking. Coxae with thin pale yellow setae; femora and tibiae with thin pale yellow setae and ventral thicker blackish setae; tibial spurs narrow, blackish; tarsi with robust blackish setae (Fig. 11D).

***Wings***. Forewing long, broad, apex rounded. Venation dense, with many crossveins. Pterostigma with four or five forked and unforked brown veinlets; membrane dark brown. Presectoral area with ~4 crossveins. RP with five branches. MA gradually curving towards hind margin in distal portion. MP-CuA area with ~3 irregular rows of cells. Anal area hind margin straight; axillary lobe inconspicuous. Forewing venation dark brown, Sc and R paler; anal veins pale brown. Forewing membrane hyaline. Hindwing broad, widest after RP fork; hind margin smoothly curved toward rounded apex. Presectoral area with three crossveins. RP with four branches. MA straight, only slightly curved. MP-CuA area with ~2 irregular rows of cells (Figs 2E, 2F, 11A). Hindwing venation dark brown, paler near wing base. Hindwing membrane hyaline, distal section with a large brown marking extending from pterostigma to cubital area, shaped as curved band gradually narrowing posteriorly, pigmentation bordering crossveins, central part of cells paler; marking fainter in males than in females (Figs 2E, 2F, 11A).

***Abdomen***. Tergites brown. Sternites brown, paler than tergites. Pleural membrane brown with large paler markings.

**Figure 12.**
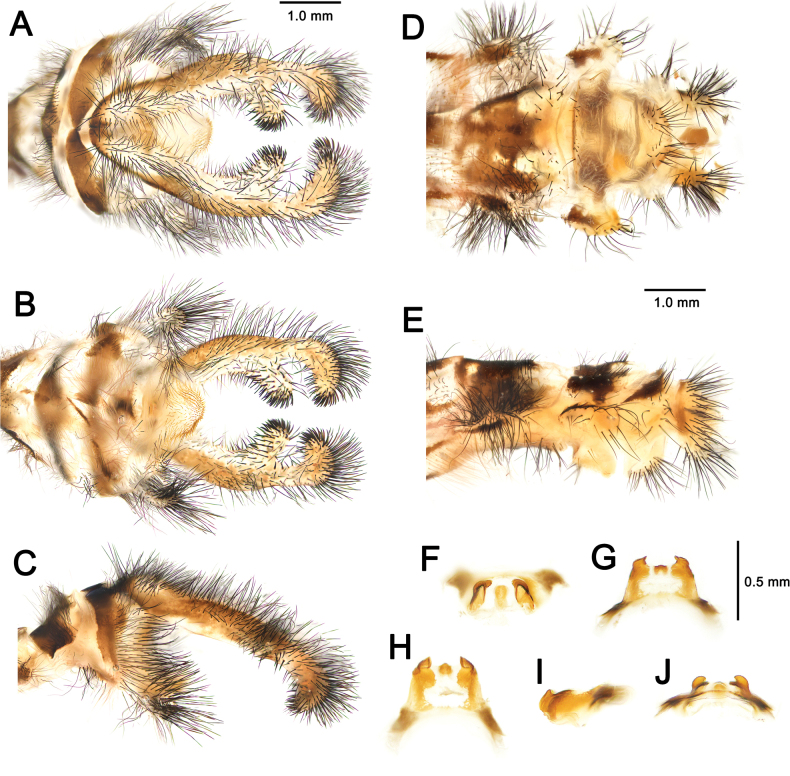
*Deleproctophylla dandizenor* Badano, Zheng, U. Aspöck & Dobosz, sp. nov., Kunar (Afghanistan). Male terminalia of holotype: **A**. Dorsal view; **B**. Ventral view; **C**. Lateral view. Female terminalia of paratype: **D**. Ventral view; **E**. Lateral view. Male genitalia of holotype: **F**. Ventral view; **G**. Anteroventral view; **H**. Caudal view; **I**. Lateral view; **J**. Dorsal view.

***Male genitalia***. Ectoproct robust, with branch at two-thirds of length and apex stout. Setae of ectoproct dense, long, dark brown, gradually longer caudally; setae on whole branch, denser, and spine-like at apex; setae on apex of ectoproct spine-like (Fig. 12A–C). Tergite 9 sub-rectangular in lateral view, with robust ventral projection; setae long and black, longer and denser on caudal margin of the sclerite and on projection. Sternite 9 with lateral lobes inconspicuous, rather small, median lobe prominent, apically rounded (Fig. 12B, C). Gx11 arch-shaped, slightly curved; gx9 strongly sclerotized, with prominent dorso-apical fold, giving to apex a hooked appearance, gp9 relatively small, lightly sclerotized forming a shallow median furrow, with short setae (Fig. 12F–J).

***Female genitalia***. Ectoproct elongated, relatively short and stout ventral projection; setae long, black (Fig. 12D, E). Tergite 9 sub-rectangular. Gx9 wider than long, prominent; setae long. Tergite 8 subrectangular. Gx8 prominent, as long as wide, with long setae. Gp8 projecting ventrally, with prominent caudal margin; setae thin (Fig. 12D, E).

##### Distribution.

Asia: Afghanistan, Pakistan (Fig. 25).

##### Etymology.

The species epitheton is derived from the name of a mountain range in the east of Afghanistan. It is a substantive and an apposition to the genus.

##### Comparative notes.

*Deleproctophylla dandizenor* is a broad-winged species, easily distinguishable from all its congeners by the narrow, curved, hindwing marking that extends from pterostigma almost to the posterior wing margin. The hindwing appears more pigmented in female in comparison to male, in which the marking is reduced to an infuscation (Fig. 2E, F). A similar difference in wing pattern is also observed in *D. tengri* sp. nov., despite the shape of markings is very different in these two species. However, due to the limited number of known specimens, it remains unclear whether these differences are attributable to sexual dimorphism or individual variation. The male of *D. dandizenor* is characterized by the robust hairy ectoproct with branch that bifurcates after mid-length, and stout apex. Moreover, the spine-like setae on the branch and on the apex of the ectoproct are rather long and slightly curved and they extend for the whole length of both structures (Fig. 12A–C). The hair-like setae on the caudal margin of tergite 9 are also diagnostic of this species. The female of *D. dandizenor* differs from the other species of the genus by the relatively stout ectoproct, with very short ventral projection and the prominent gx8 and gp8 (Fig. 12D, E).

#### Deleproctophylla dusmeti

Taxon classificationAnimaliaNeuropteraMyrmeleontidae

(Navás, 1914)

B7B52E33-475C-580D-9865-317C008BD42E

**Figure 13.**
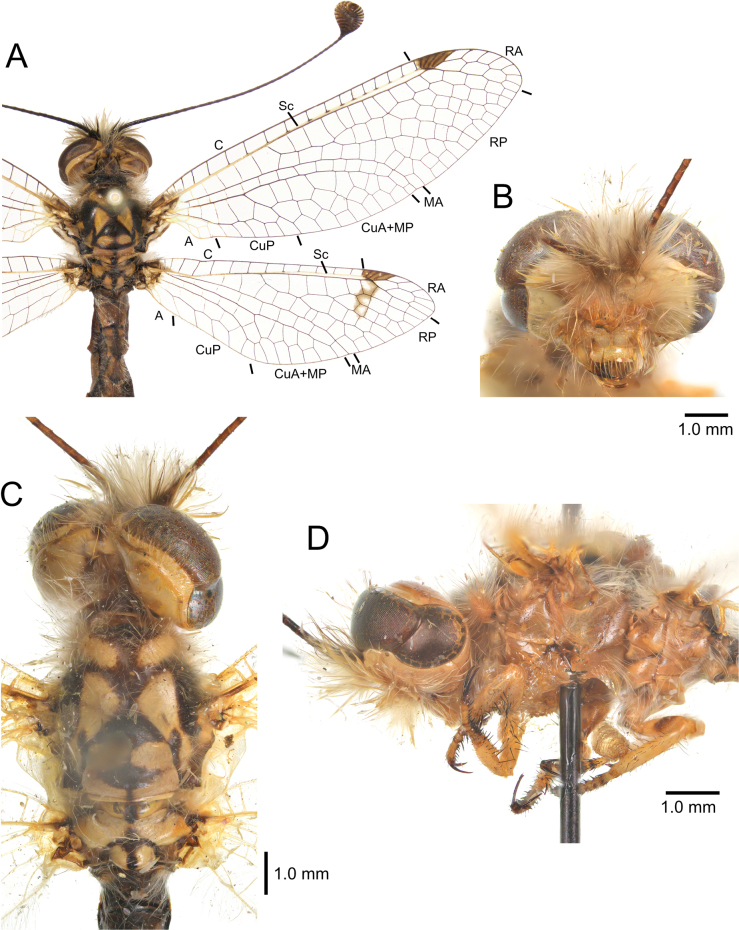
*Deleproctophylla dusmeti* (Navás, 1914). **A**. Habitus, Saint-Paul-et-Valmalle (France); **B**. Head, frontal view, Toulon (France); **C**. Head and thorax, dorsal view, Toulon (France); **D**. Head and thorax, lateral view, Besse-sur-Issole (France). Abbreviations: C costa Sc subcosta RA radius anterior RP radius posterior MA media anterior MP media posterior CuA cubitus anterior CuP cubitus posterior A anal veins.

**Figure 14.**
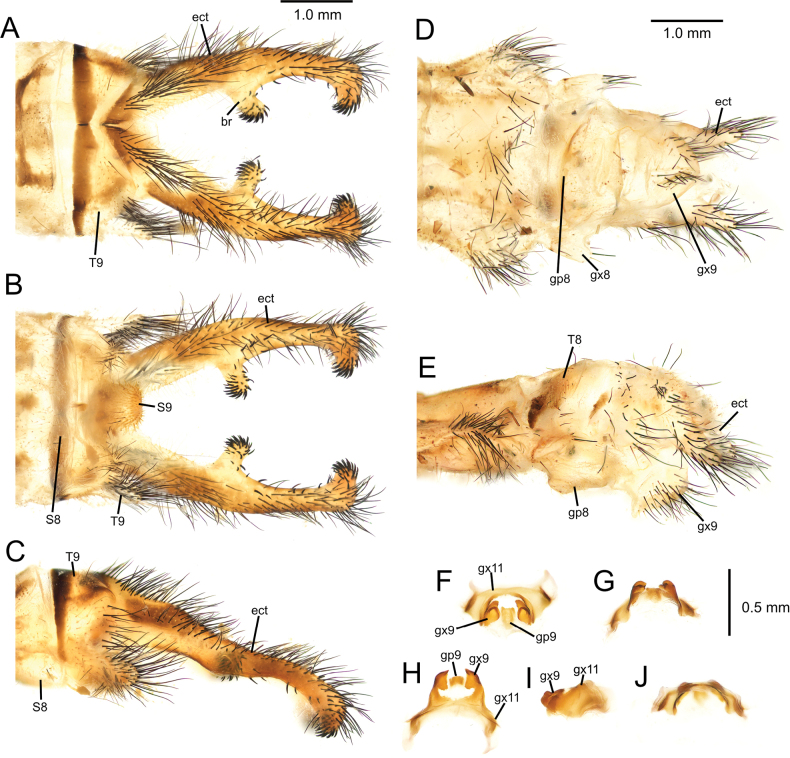
*Deleproctophylla dusmeti* (Navás, 1914), Madrid (Spain). Male terminalia: **A**. Dorsal view; **B**. Ventral view; **C**. Lateral view. Female terminalia: **D**. Ventral view; **E**. Lateral view. Male genitalia: **F**. Ventral view; **G**. Anteroventral view; **H**. Caudal view; **I**. Lateral view; **J**. Dorsal view. Abbreviations: ect ectoproct br branch of ectoproct T8 tergite 8 S8 sternite 8 gx8 gonocoxite 8 gp8 gonapophysis 8 T9 tergite 9 S9 sternite 9 gx9 gonocoxite 9 gp9 gonapophysis 9 gx11 gonocoxite 11.

Figs 2G, 2H, 4G, 4H, 5C, 5D, 13, 14

Theleproctophylla dusmeti Navás, 1914: 57 (type locality: Spain: Madrid, Montarco; lectotype in MNCN). Navás 1915a: 68 (Theleproctophylla). Navás 1915b: 461 (Theleproctophylla). Bohigas and Sánchez 1917: 311 (Theleproctophylla). Navás 1920: 7 (Deleproctophylla). Escribano 1921: 365 (Theleproctophylla). Monserrat 1978: 182 (Deleproctophylla). H. Aspöck et al. 1980: 316 (with some questionable distribution, Deleproctophylla). Monserrat 1982: 70 (Deleproctophylla). H. Aspöck and Hölzel 1996: 78 (with some questionable distribution, Deleproctophylla). Sziráki 1998: 61 (with some questionable distribution, Deleproctophylla). H. Aspöck et al. 2001:302 (Deleproctophylla); Whittington 2002: 377 (with some questionable distribution, Deleproctophylla). Canbulat and Kiyak 2004: 114 (probably misidentification, Deleproctophylla). Canbulat 2007: 36 (probably misidentification, Deleproctophylla). Dobosz and Ábrahám 2007: 24 (probably misidentification, Deleproctophylla). Koçak and Kemal 2012: 17 (probably misidentification, Deleproctophylla). Monserrat et al. 2012: 38 (Deleproctophylla). Monserrat 2013: 298 (Deleproctophylla). Monserrat et al. 2014: 153 (Deleproctophylla). Badano and Pantaleoni 2014: 301 (Deleproctophylla). Aistleitner 2019: 123 (with some questionable distribution, Deleproctophylla). Hassan et al. 2019: 513 (questionable distribution, Deleproctophylla). Aistleitner and Lencina 2020: 102 (Deleproctophylla). Monnerat and Ábrahám 2020: 144 (questionable distribution, Deleproctophylla). Hassan and Liu 2021: 416 (questionable distribution, Deleproctophylla). Poggi 2024: 9 (Deleproctophylla).

##### Examined material.

**Type specimen. *Paralectotype***: • Granada, 8.VII.01, Typus, *Theleproctophylla dusmeti* Navás SJ det., *Theleproctophylla dusmeti* Navás, 1914, Paralectotype, J. Legrand det, 1 ♀ (MNHN) (Fig. 24A, B).

##### Other specimens.

• France: Alpes Maritimes, Villeneuve Loubet, 43°39'44.08"N, 3°40'36.14"E, 16.VII.2011, D. Badano, 4 ♂ 5 ♀ (DB); • France: Bouches-du-Rhône, Eygalières, Le Mas de Montfort, m 170, 43°44'48.7"N, 04°55'17.7"E, 26.VI.2019, 2 ♂ (DB); • France: Besse/Var, 250 m, 18.VII.1964, Bilek, 1 ♂ 1 ♀ (HUAC); • France: Hérault, Gigean, 26–29.VI.2014, A. Prost, 2 ♂ 1 ♀ (MDC); • France: Hérault, Prades Le Lez, 43°40'58.78"N, 3°52'33.30"E, 11.VII.2011, D. Badano, 3 ♀ (DB); • France: Hérault, St. Paul et Valmalle, 43°36'36.14"N, 3°40'36.14"E, 26.VI.2011, D. Badano, 2 ♂ 1 ♀ (DB); • same locality, 29.VI.2011, D. Badano, 2 ♀ (DB); • same locality, 5.VII.2011, D. Badano, 4 ♂ 1 ♀ (DB); • same locality, 10.VII.2011, D. Badano, 3 ♂ 3 ♀ (DB); • France: Rochefort-du-Gard, 18.07.2002, J. Bard, 1 ♂ (SCM); • Portugal: Algarve, Odelouca, 14.VI.1970, C. and A. Jeekel, 1 ♀ (RMNH); • Spain: Aragona, Sena, 17.VII.1923, L. Navás, 1 ♂ 1 ♀ (MSNG); • Spain: Granada, 8.VII.1901, 1 ♀ (MNHN); • Spain: Madrid, Coll. Lacroix, 1 ♂ 2 ♀ (MNHN); • Spain: Zaragoza, Pina de Ebro, 05.07.1991, V. Redondo, 1 ♂ (SCM).

##### Diagnostic characters.

Metafemur with brown marking; forewing unmarked; hindwing subtriangular, posterior margin angled; hindwing with marking below pterostigma; male ectoproct with branch at mid-length; setae on branch only at tip; setae on branch and tip of ectoproct stout and curved.

##### Redescription.

***Size*** (mm, based on 5 specimens). Head + thorax length 7.8–8.3; antenna length 14.1–16.5; fore wing length 19.7–23.8; fore wing width 5.6–6.4; hindwing length 15.3–18.4; hindwing width 4.7–5.8.

***Head***. Occiput yellowish brown; setae dense, long, pale yellow. Vertex yellowish brown; setae dense, long, pale yellow (Fig. 13C). Para-ocular band pale yellow. Frons yellowish brown; setae dense, long, pale yellow. Clypeus and labrum yellowish brown; setae long, pale (Fig. 13B). Mandible yellowish brown, dark brown apically. Labium and palp yellowish brown. Antenna with pedicel with dense pale and dark setae, flagellomeres brown, nodes slightly darker; club brown, paler anteriorly (Fig. 13A).

***Thorax***. Cervical sclerite yellowish brown. Pronotum brown; setae long, pale yellow. Mesonotum brown with yellowish brown markings: an anterior stripe-like pair, a median triangular pair, and a posterior rounded pair; mesoscutellum dark brown with a pair of yellowish rounded markings and posterior margin; setae pale yellow. Metanotum largely yellowish brown, medially dark brown; setae pale yellow (Fig. 13C). Pleurae yellowish brown; setae pale yellow (Fig. 13D).

***Legs***. Pro- and mesothoracic legs yellowish brown, tarsi slightly darker. Metathoracic leg yellowish brown, dorsal side of metafemur with dark brown marking. Setae of coxae pale yellow setae; setae of femora and tibiae thin and pale yellow dorsally, thicker and blackish ventrally; tibial spurs narrow; setae of tarsi blackish (Fig. 13D).

***Wings***. Forewing long, apex rounded. Pterostigma with four to five forked and unforked brown veinlets; membrane brown. Presectoral area with five crossveins. RP with five branches. MA gently toward hind margin. MP-CuA area with ~3 irregular rows of cells. Anal area hind margin straight; axillary lobe slightly pronounced. Forewing venation largely dark brown, paler toward wing base; Sc and R pale brown; anal veins pale brown. Forewing membrane hyaline (Figs 2G, 2H, 4G, 4H, 13A). Hindwing broad, subtriangular, widest after RP fork; hind margin angled, gradually narrowing toward rounded apex. Presectoral area with three crossveins. RP with four branches. MA largely straight, slightly curved distally. MP-CuA area with ~2 irregular rows of cells. Hindwing venation dark brown, paler near wing base. Hindwing membrane hyaline, distal section with a rather large brown marking extending from pterostigma across to second branch of RP (Figs 2G, 2H, 4G, 4H, 13A).

***Abdomen***. Tergites pale brown, with caudal pairs of yellowish markings. Sternites pale brown, paler than tergites. Pleural membrane brown with large paler markings.

***Male genitalia***. Ectoproct narrow, with branch at mid-length. Setae of ectoproct long, dark brown; setae on branch limited to tip, dense, black, stout, curved, and spine-like; setae only on tip of ectoproct, black, stout, curved, and spine-like (Fig. 14A–C). Tergite 9 sub-rectangular in lateral view, with ventral projection; setae long and black, longer and denser on projection. Sternite 9 with paired lateral and a single median lobe; setae thin, spine-like on median lobe (Fig. 14B, C). Gx11 arch-shaped, projecting caudally and slightly curved downward in lateral view; gp11 lightly sclerotized, forming a median furrow, with minute setae; gx9 widely set apart, strongly sclerotized, dorsally folded (Fig. 14F–J).

***Female genitalia***. Ectoproct elongated, projecting ventrally; setae long, black. Tergite 9 sub-rectangular. Gx9 wider than long; setae long (Fig. 14D, E). Tergite 8 subrectangular. Gx8 wider than long, with long setae. Gp8 projecting ventrally; setae thin (Fig. 14D, E).

**Larva**. See Badano and Pantaleoni (2014) (Fig. 5C, D).

##### Distribution.

Europe: France, Portugal, Spain (H. Aspöck et al. 1980; Rehfeldt 1989; H. Aspöck et al. 2001; Monserrat et al. 2014; Oswald 2025) (Fig. 25).

##### Comparative notes.

*Deleproctophylla dusmeti* may be difficult to distinguish from its two congeners in western Europe, *D. australis* and *D. bleusei*. The wing patterns traditionally used to differentiate these species are unreliable and exhibit high individual variation (Fig. 4). The diagnostic characters of *D. dusmeti* include a triangular-shaped hindwing (in contrast to the elongated hindwing of *D. australis*) and a male ectoproct branch that protrudes at mid-length (whereas in *D. australis* and *D. bleusei* the branch arises after mid-length) (Figs 13A, 14A).

##### Remarks.

*D. dusmeti* has been reported from several Asian countries, including Azerbaijan, Turkey, and Pakistan (H. Aspöck et al. 2001; Canbulat and Kiyak 2004; Canbulat 2007; Dobosz and Ábrahám 2007; Koçak and Kemal 2012; Hassan et al. 2019; Monnerat and Ábrahám 2020; Hassan and Liu 2021; Oswald 2025). However, the examination of the specimens assigned to the “*dusmeti*-like form” (see H. Aspöck et al. 1980) suggest that these instead belong to *D. australis*, representing individuals without forewing marking, a common trait in eastern populations of this species. The vague report from Pakistan (Hassan et al. 2019; Hassan and Liu 2021; Oswald 2025), which lacks any locality information cannot be verified at present. It may actually refer to *D. dandizenor* sp. nov., another species with unmarked forewing. The current observations suggest that *D. dusmeti* is exclusively a western European species. *Deleproctophylla dusmeti* is typically distinguished from *D. australis* by the absence of forewing spots. However, some populations along the southeastern coast of France include individuals with marked forewings mixed with unmarked ones (Fig. 4H). The study of genital morphology and species delimitation analyses confirm that these specimens belong to *D. dusmeti* (Fig. 1). These spotted individuals are likely the source of records of *D. australis* in southern France, despite this species is absent from the French mainland (though it is present in Corsica). The distributions of *D. dusmeti* and *D. australis* do not overlap, being separated by a relatively small geographic gap along the northern Tyrrhenian coast of Italy (Fig. 25).

#### Deleproctophylla gelini

Taxon classificationAnimaliaNeuropteraMyrmeleontidae

(Navás, 1919)

F401A582-E6BF-5788-88B5-DC172056FE51

Figs 3A, 3B, 15, 16

Theleproctophylla gelini Navás, 1919: 21 (type locality: Morocco: Safi; holotype in MNHN but lost at present). Navás 1930: 121 (Theleproctophylla). H. Aspöck and Hölzel 1996: 78 (Deleproctophylla). H. Aspöck et al. 2001:302 (Deleproctophylla). Whittington 2002: 377 (Deleproctophylla). Ábrahám and Mészáros 2004: 333 (Deleproctophylla). Faucheux 2006: 204 (Deleproctophylla). Faucheux et al. 2012: 171 (Deleproctophylla). Ábrahám 2017: 126 (Deleproctophylla).

##### Examined material.

• Morocco: Agadir, 27.V–6.VI.1973, C. and A. Jeekel, 2 ♀ (RMNH); • Morocco: Bou Tazzert, Pres Mogadoa, 1928, R. Benoist. 2 ♂, 1 ♀, 2 ex. (MNHN); • Morocco: Tamri, 30°42'31.9"N, 09°51'26.1"W, 15 m, 30.06.2008, L. Ábrahám, L. Bognár and L. Nagy, 5 ♂♂ 1 ♀ (SCM); • Morocco: Tamri, 30°42'31.9"N, 09°51'26.1"W, 15 m, 30.06.2008, L. Ábrahám, L. Bognár and L. Nagy, 1 ♂ 1 ♀ (USMB); • Morocco: Atlantic Plain, 4 km W Agoudi n’Aït Amar 30°42'31.3"N, 9°51'26.2"W, 10 m, 27.06.2024, W. Szczepański, 4 ♂♂ 2 ♀♀ (USMB);

##### Diagnostic characters.

Vertex as long as wide; metafemur without brown marking; forewing extensively marked; hindwing broad, posterior margin curved; forewing with complex pattern: brown marking at base, subcostal area brown, brown marking at RP bifurcation, and large brown marking from pterostigma to first branch of RP; hindwing with a similar pattern: brown marking at base, subcostal area brown, brown marking at RP bifurcation, and large brown marking from pterostigma to posterior margin; male ectoproct bent inward, with branch at mid-length and protruding dorsally; setae on branch only at tip; setae on tip of ectoproct robust and relatively long; male with gx11 straight, broad, incapsulating gx9; gx9 without apical fold; female ectoproct long; gx8 and gp8 not prominent.

##### Redescription.

***Size*** (mm, based on 3 specimens). Head + thorax 7.4–9; antenna length 14.9–16.9; fore wing length 21–24.9; fore wing 6.2–7.3; hindwing 16.1–20.5; hindwing width 5.2–6.1.

**Figure 15.**
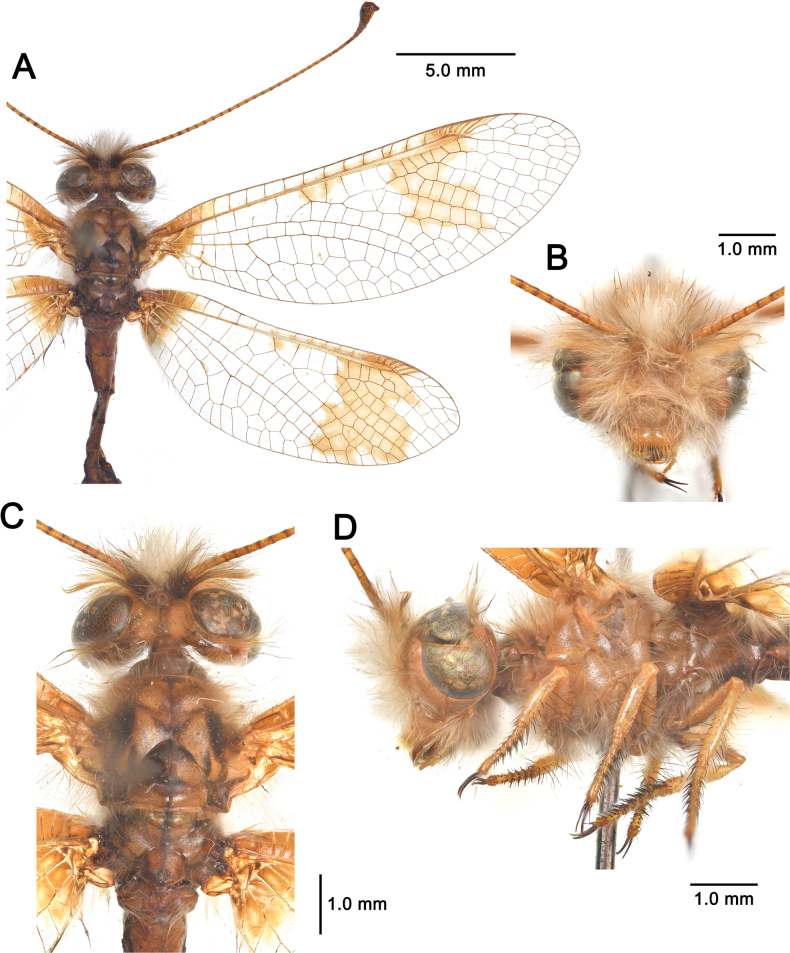
*Deleproctophylla gelini* (Navás, 1919), Guigou (Morocco). **A**. Habitus; **B**. Head, frontal view; **C**. Head and thorax, dorsal view; **D**. Head and thorax, lateral view.

***Head***. Vertex nearly as long as wide, yellowish brown; setae dense, long, pale yellow (Fig. 15C). Para-ocular band pale yellow. Frons yellowish brown; setae dense, long, pale yellow. Clypeus and labrum yellowish brown; setae long, pale (Fig. 15B). Mandible yellowish brown, dark brown apically. Labium and palp yellowish brown. Antenna with pedicel with dense pale and dark setae, flagellomeres pale brown, nodes dark brown; club dark brown, paler anteriorly (Fig. 15A).

***Thorax***. Cervical sclerite yellowish brown. Pronotum brown; setae long, pale yellow. Mesonotum yellowish brown gradually darker medially and along sclerites margins. Metanotum yellowish brown, medially darker; setae pale yellow (Fig. 15C). Pleurae yellowish brown; setae pale yellow (Fig. 15D).

***Legs***. Pro- and mesothoracic legs yellowish brown, including tarsi. Metathoracic leg yellowish brown, unmarked. Setae of coxae pale yellow setae; setae of femora and tibiae thin and pale yellow dorsally, thicker and blackish ventrally; tibial spurs narrow; setae of tarsi blackish (Fig. 15D).

***Wings***. Forewing broad, apex rounded. Pterostigma with ~5 forked and unforked brown veinlets; membrane brown. Presectoral area with five crossveins. RP with ~5 branches. MA gently toward hind margin. MP-CuA area with ~3 irregular rows of cells. Anal area hind margin straight; axillary lobe slightly pronounced. Forewing venation largely pale brown. Forewing membrane largely hyaline with a complex brown pattern: base of wing brown, apical costal crossveins with brown shade at base, subcostal area brown, brown marking at RP branching, apical portion of wing with a large brown marking extending from subcostal area and pterostigma to first branch of RP (Figs 3A, 3B, 15A). Hindwing broad, widest at RP fork; hind margin rounded, smoothly curved toward rounded apex. Presectoral area with three crossveins. RP with five branches. MA largely straight, slightly curved distally. MP-CuA area with ~2 irregular rows of cells. Hindwing venation pale brown, Hindwing membrane hyaline, with a complex brown pattern: base of wing brown, apical costal crossveins with brown shade at base, subcostal area brown, brown marking at RP branching, apical portion of wing with a large brown marking extending from subcostal area and pterostigma to posterior margin of wing (Figs 3A, 3B, 15A).

***Abdomen***. Tergites pale brown. Sternites pale brown, paler than tergites. Pleural membrane brown.

**Figure 16.**
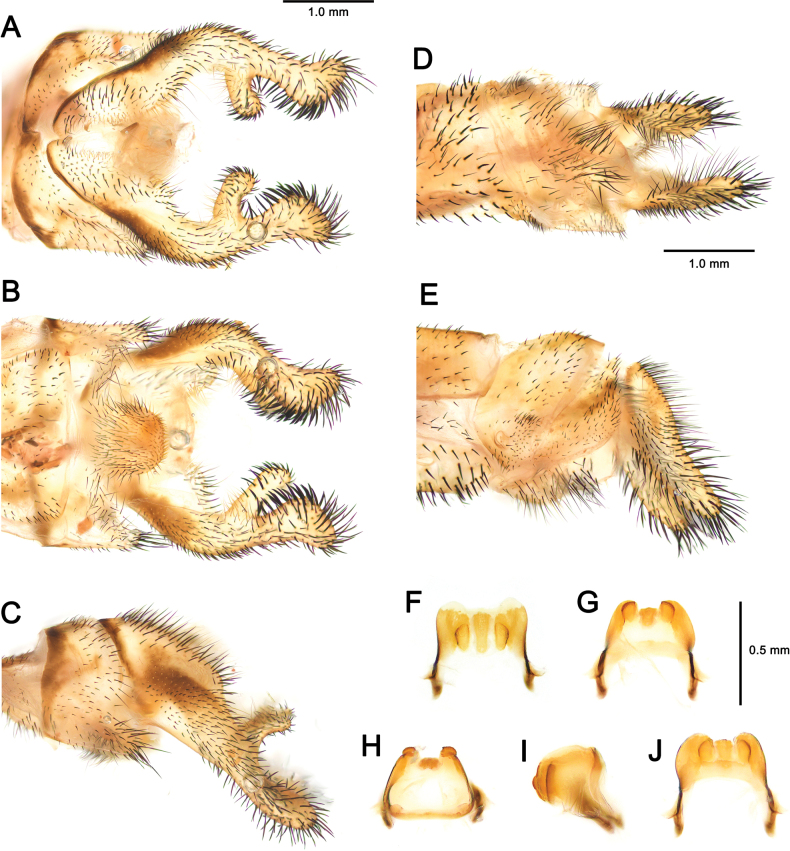
*Deleproctophylla gelini* (Navás, 1919), Guigou (Morocco). Male terminalia: **A**. Dorsal view; **B**. Ventral view; **C**. Lateral view. Female terminalia: **D**. Ventral view; **E**. Lateral view. Male genitalia: **F**. Ventral view; **G**. Anteroventral view; **H**. Caudal view; **I**. Lateral view; **J**. Dorsal view.

***Male genitalia***. Ectoproct robust, strongly bent inward, with branch slightly beyond mid-length. Branch projecting dorsally, broad, and relatively thick, progressively widening at apex. Apex of ectoproct club-shaped expanding internally. Setae of ectoproct short, dark brown; setae on branch sparse, those on apical section, black, stout, and curved; setae on tip of ectoproct progressively denser caudally, black, stout, curved, and spine-like on the internal side (Fig. 16A–C). Tergite 9 trapezoid in lateral view, with broad ventral projection; setae long and black, longer and denser on projection (Fig. 16B, C). Sternite 9 without paired lateral lobes, being composed only by the median lobe; setae thin, spine-like lobe. Gx11 arch-shaped, projecting caudally, broad and incapsulating gx9, straight in lateral view; gp11 lightly sclerotized, elongated, with a shallow median furrow, with minute setae; gx9 widely set apart, strongly sclerotized, flattened, without dorsal fold (Fig. 16F–J).

***Female genitalia***. Ectoproct strongly elongated, projecting ventrally; setae long, black (Fig. 16D, E). Tergite 9 sub-rectangular. Gx9 wider than long; setae long. Tergite 8 subrectangular. Gx8 not prominent, with long setae. Gp8 not prominent; setae thin. Sternite 7 with sparse stout setae (Fig. 16D, E).

##### Distribution.

Africa: Morocco (H. Aspöck et al. 2001; Ábrahám and Mészáros 2004; Oswald 2025).

##### Comparative notes.

Among all *Deleproctophylla* species, *D. gelini* is the easiest to identify due to its unique fore- and hindwing pattern (Ábrahám and Mészáros 2004) (Fig. 3A, B). Additionally, this species differs from all congeners in both male and female genitalia, which are characterized by several distinctive characters (Ábrahám and Mészáros 2004) (Fig. 16). The male ectoproct is stout and distinctly bent inward and the branch arises dorso-caudally at mid-length. The branch stacks on the ectoproct, instead of being directed inward, as seen in all other species. Furthermore, the sternite 9 of the male lacks the lateral lobes found in other species (Fig. 16A–C). The gx9+11 complex is also different being, shaped like a capsule. The Gx11 is broad, not curved downward, and encases the gx9, which lacks the apical dorsal fold present in all other species (Fig. 16F–J). The female of *D. gelini* is characterized by an elongated ectoproct projecting ventrally and reduced gx8 (Fig. 16D, E).

#### Deleproctophylla tengri

Taxon classificationAnimaliaNeuropteraMyrmeleontidae

Zheng, Badano, H. Aspöck & Liu sp. nov.

67F1EA64-EACE-5BEF-8317-C0FA5BB2A1AA

https://zoobank.org/885D76FC-EBFA-4850-B43A-78355B359BCA

Figs 3C, 3D, 17–21

##### Type material.

***Holotype*** • China: Xinjiang, Shihezi [石河子], Maangou Village [马鞍沟村], Mt. Jiangjunshan [将军山], 650 m, 6.VI.2020, Haitian Song, 1 ♂ (IZCAS). ***Paratypes*** • Same locality as holotype, 18.VII.2022, Rui Wang, 1 ♀ (IZCAS); • same locality as holotype, 14.VII.2025, Yuchen Zheng and Rui Wang, 1 ♂ 1 ♀ (CAU); • Kazakhstan: Alma-Ata (present Almaty), 1 ♀ (NHMW); • Kyrgyzstan: Narinskaya Obl. NE Naryn, Aral, 1500 m, 10.VII.2000, Vladimir Dolin, 1♂ (HUAC); • Kyrgyzstan: 5 km NE of Ugut, 41°25.561'N, 74°52.358'E, 1616 m, 09.07.2013, L. Ábrahám and S. Ilniczky, 1 ♂ 2 ♀♀ (SCM); • Kyrgyzstan: 2 km S of Kala, 41°26.669'N, 72°12.873'E, 789 m, 03.07.2013, L. Ábrahám and S. Ilniczky, 6 ♀♀ (SCM); • Kyrgyzstan: Jalal-Abad Region, ca 7 km N of Tash-Kömür, 41°25'N, 72°15'E, 660 m, 20–21.06.2003, R. Dobosz, 1 ♀ (Phot. 1) (USMB); • Tajikistan: Gorno-Badakhshan, S. side Saghirdasht Pass 10 km N. of Kala-i Khumb, 38°32'51.7"N, 70°48'1.6"E, 1850 m, T. and W. Garrevoet, 1 ♀ (RMNH).

##### Larval material.

China: Xinjiang, Shihezi, 5 km south of the Shihezi City, 686 m, 20.V.2025, Ziyang Ni (ZCAU).

##### Diagnostic characters.

Metafemur unmarked; forewing with acute apex, hyaline, without marking; hindwing subtriangular, posterior margin angled; hindwing marking generally as shade along crossveins, extending on RP; male ectoproct median branch basally wide, gradually bifurcating, subtriangular shape.

##### Description.

***Size*** (mm, based on 2 specimens). Head + thorax length 6.9; antenna length 11.22–14.15; fore wing length 20.1–21.2; fore wing width 6.1–6.5; hindwing length 15–16; hindwing width 5.7–6.2.

**Figure 17.**
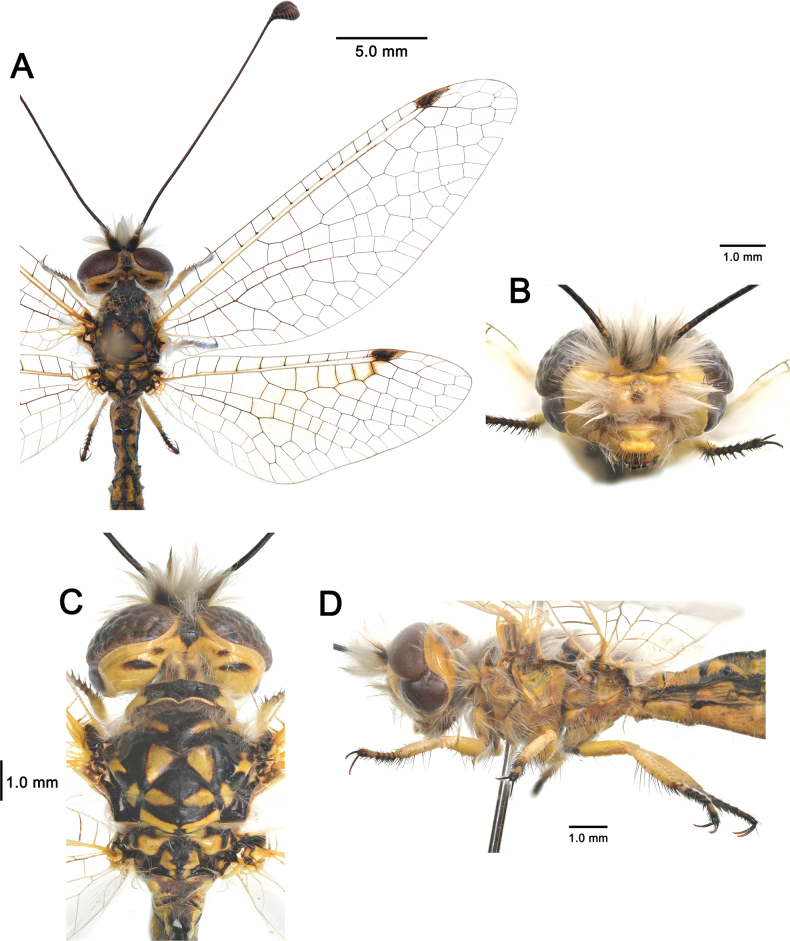
*Deleproctophylla tengri* Zheng, Badano, H. Aspöck & Liu, sp. nov., adult, Xinjiang (China). **A**. Habitus, paratype female; **B**. Head, frontal view, holotype male; **C**. Head and thorax, dorsal view, holotype male; **D**. Head and thorax, lateral view, holotype male.

***Head***. Vertex dark brown; setae dense, long, pale (Fig. 17C). Para-ocular band pale yellow. Frons yellow, medially with a dark brown spot; setae dense, long, pale. Clypeus and labrum yellow; setae long, pale (Fig. 17B). Mandible yellow, transitioning to dark brown apically. Labium and palp yellow. Antenna pedicel with dense pale and dark setae, flagellomeres generally dark brown; club basally dark brown, gradually transitioned to pale yellow to the tip (Fig. 17A).

***Thorax***. Cervical sclerite yellow, with a pair of dark markings. Pronotum black, lateral margin yellow; setae long, pale. Mesonotum black with yellow markings: an anterior transversal pair, a median triangular pair and two lateral pairs; mesoscutellum mostly black, medially with a pair of yellowish markings, posterior margin yellow (Fig. 17C). Pleurae pale yellow (Fig. 17D).

***Legs***. Pro- and mesothoracic legs pale yellow, tarsi each generally pale yellow and distally dark brown; hind tarsus mostly dark brown. Coxae with thin pale setae; femora and tibiae with thin pale setae and ventral thicker blackish setae; tibial spurs narrow, blackish; tarsi with robust blackish setae (Fig. 17D).

***Wings***. Forewing long, apex acute. Pterostigma with three to four forked and unforked brown veinlets; membrane brown. Presectoral area with five to six crossveins. RP with four branches. MA gradually curving toward hind margin in distal portion. MP-CuA area with ~3 irregular rows of cells. Anal area hind margin straight; axillary lobe slightly pronounced. Forewing venation largely brown, paler toward wing base; Sc pale yellow, paler toward distal part; R pale yellow, darker toward distal part. Forewing membrane hyaline (Figs 3C, 3D, 17A). Hindwing subtriangular, broadest at RP fork; hind margin angular curved narrowing toward rounded apex. Presectoral area with two crossveins. RP with three branches. MA largely straight, slightly curved toward hind margin distally. MP-CuA area with ~2 irregular rows of cells. Hindwing venation brown, paler near wing base and distally. Hindwing membrane hyaline, hindwing marking generally as shade along crossveins, extending on RP (Figs 3C, 3D, 17A).

***Abdomen***. Tergites black, each with a caudal pair of yellowish markings. Sternites yellow. Pleural membrane brown with large paler markings.

**Figure 18.**
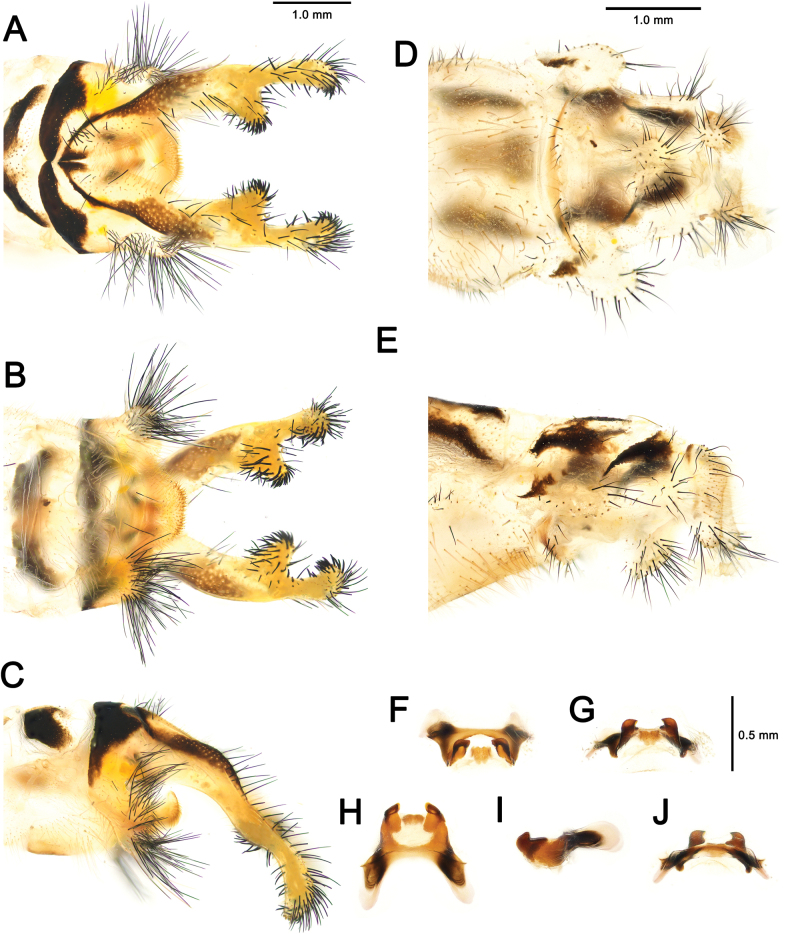
*Deleproctophylla tengri* Zheng, Badano, H. Aspöck & Liu, sp. nov., adult, Xinjiang (China). Male terminalia of holotype: **A**. Dorsal view; **B**. Ventral view; **C**. Lateral view. Female terminalia of paratype: **D**. Ventral view; **E**. Lateral view. Male genitalia of holotype: **F**. Ventral view; **G**. Anteroventral view; **H**. Caudal view; **I**. Lateral view; **J**. Dorsal view.

***Male genitalia***. Ectoproct narrow, with branch in apical half, basally wide, gradually bifurcating, subtriangular shape. Setae of ectoproct long, dark brown; setae on branch limited to tip, dense, black, stout, curved, and spine-like; setae only on tip of ectoproct, black, stout, curved, and spine-like (Fig. 18A–C). Tergite 9 sub-rectangular in lateral view, with ventral projection; setae long and black, longer and denser on projection. Sternite 9 wide trapezoid in ventral view; setae thin, spine-like on median part (Fig. 18B, C). Gx11 arch-shaped, external tip rounded, slightly curved downward in lateral view; gp11 lightly sclerotized forming a shallow median furrow, with short setae; gx9 strongly sclerotized, with upward fold, widely hook-like in lateral view (Fig. 18F–J).

***Female genitalia***. Ectoproct elongated; setae long, black. Tergite 9 sub-rectangular. Gx9 longer than wide; setae long. Tergite 8 subrectangular. Gx8 wider than long, with long setae. Gp8 flatted; setae thin (Fig. 18D, E).

##### Diagnosis of 3^rd^ instar larva.

Dorsal head yellowish brown, with many dark brown spots, spots on the middle part relatively sparse, anteromedially with a dark brown marking. Dorsal segments 1–7 medially with a pair of short dark brown stripes, laterally scattered with irregular dense dark brown markings (Fig. 20).

##### Description of 3^rd^ instar larva.

***Size*** (mm). Body length (excluding mandible): 15.74; head length: 3.83; head width: 4.07; mandible length: 4.06.

***Head***. Wider then long, laterally with short setae (Fig. 21A, B). Clypeo-labrum generally brown, concave internally; anterior margin covered with some dolichasters. Dorsal side of head capsule yellowish brown, with many dark brown spots, spots on the middle part relatively sparse, anteromedially with a dark brown marking; head ventrally yellowish brown, with many short dolichasters, pore developed. Ocular tubercle prominent and large, apical part with a tiny projection. Antennae brown (Fig. 21A, B). Mandible nearly as long as head width; dorsal mandible basally pale brown, gradually darkening to the distal part; ventral mandible generally dark brown. Mandible basally with three or four pseudo-teeth and three interdental mandibular setae; second tooth largest and close to the third tooth; first tooth longer than third tooth; two pseudo-teeth between first and second teeth (Fig. 21A, B).

***Thorax***. Laterally with short setae. Pronotum generally pale pink, laterally pale yellow, scattered with some dark dots, posterolaterally with a pair of black markings, covered with tiny dark dolichaster. Mesothorax generally pale yellow, with spiracles present on short stout black sclerotized tubercle, lateral margin dark brown. Meso- and metathoracic setiferous processus scolus-like, distally constrict. Anterior mesothoracic setiferous processus longer than the posterior pair (Fig. 21C).

***Legs***. Pale yellowish brown, densely covered by short setae.

***Abdomen***. Pale yellow. Dorsal segments 1–7 medially with a pair of short dark brown stripes, laterally scattered with irregular dense dark brown markings. Each ventral segments 1–7 pale, laterally scattered with many dark spots, ventral segments 2–7 medially with a dark spot. Each segment 1–7 with a pair of dorsal scolus-like processus and a pair of ventral short scolus-like processus. Abdominal segment 9 pale yellow, long trapezoid, twice as long as wide, developed rastra with four pairs of digging setae (Fig. 21D).

##### Distribution.

Asia: China (Xinjiang); Kazakhstan (Almaty Region); Kyrgyzstan (Jalalabad and Narinsky Region); Tajikistan (Gorno-Badakhshan Autonomous Region) (Fig. 25).

##### Etymology.

The species name tengri is derived from the Turkic word “tengri,” meaning sky or heaven. The species was found in the surrounding area of the Tianshan Mountains (also known as the Tengri Tagh Mountains), a mountain range whose name, across various language families, signifies “Mountains of sky or heaven”. Thus, the name tengri reflects both the geographical origin of the species and the celestial meaning of the term in Turkic languages. The species epitheton is a substantive and an apposition to the genus.

##### Comparative notes.

*Deleproctophylla tengri* can be readily distinguished from the congeners by the median branch of the male ectoproct is subtriangular and distinctly broad at the base (Fig. 18A–C). The larva of *D. tengri* sp. nov. exhibits all the diagnostic larval features of the genus and differs only slightly from the known congeners *D. australis* and *D. dusmeti* (Figs 5, 20, 21). The main distinguishing feature of *D. tengri* compared to its western counterparts is the paler head capsule, which is distinctly mottled dark brown dorsally and paler ventrally (Fig. 21A, B). In contrast, the larvae of *D. australis* and *D. dusmeti*, which are virtually indistinguishable from each other, are characterized by a uniformly dark head capsule (Fig. 5).

##### Biology.

**Figure 19.**
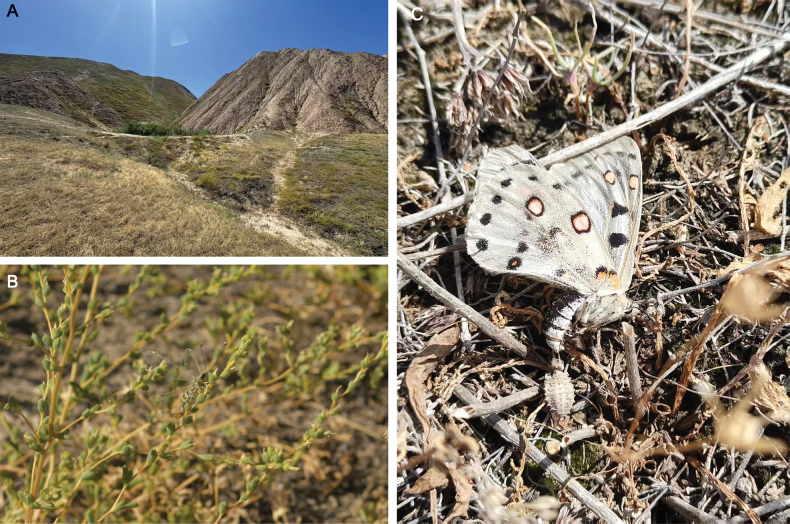
*Deleproctophylla tengri* Zheng, Badano, H. Aspöck & Liu, sp. nov., live specimens, Xinjiang (China). **A**. Habitat, Mount Jiangjunshan (Shihezi); **B**. Female adult, paratype, Mount Jiangjunshan (Shihezi); **C**. Larva, feeding on a *Parnassius apollonius* (Eversmann, 1847). **A, B**. Photos by YZ; **C**. Photo by Ziyang Ni.

*Deleproctophylla tengri* inhabits arid hills (Fig. 19A, B). The larva of this new species was observed preying on a *Parnassius apollonius* Eversmann, 1847 (Lepidoptera: Papilionidae) on a hillside meadow (Fig. 19C).

**Figure 20.**
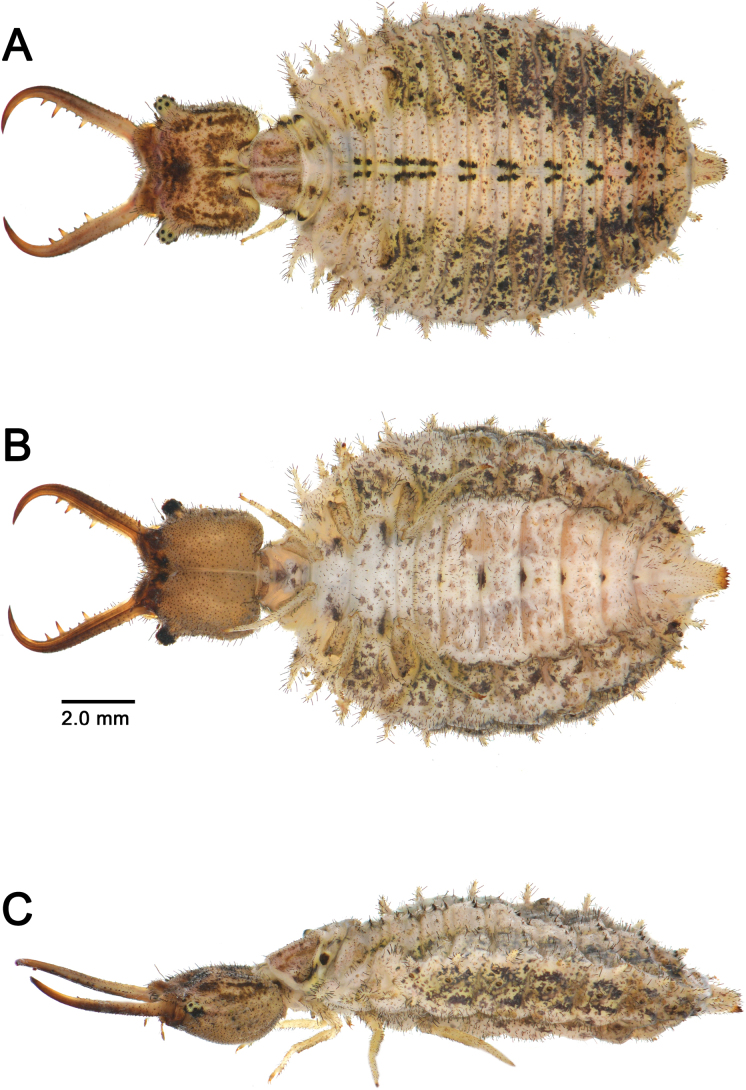
*Deleproctophylla tengri* Zheng, Badano, H. Aspöck & Liu, sp. nov., 3^rd^ instar larva, paratype Xinjiang (China). **A**. Dorsal view; **B**. Ventral view; **C**. Lateral view.

**Figure 21.**
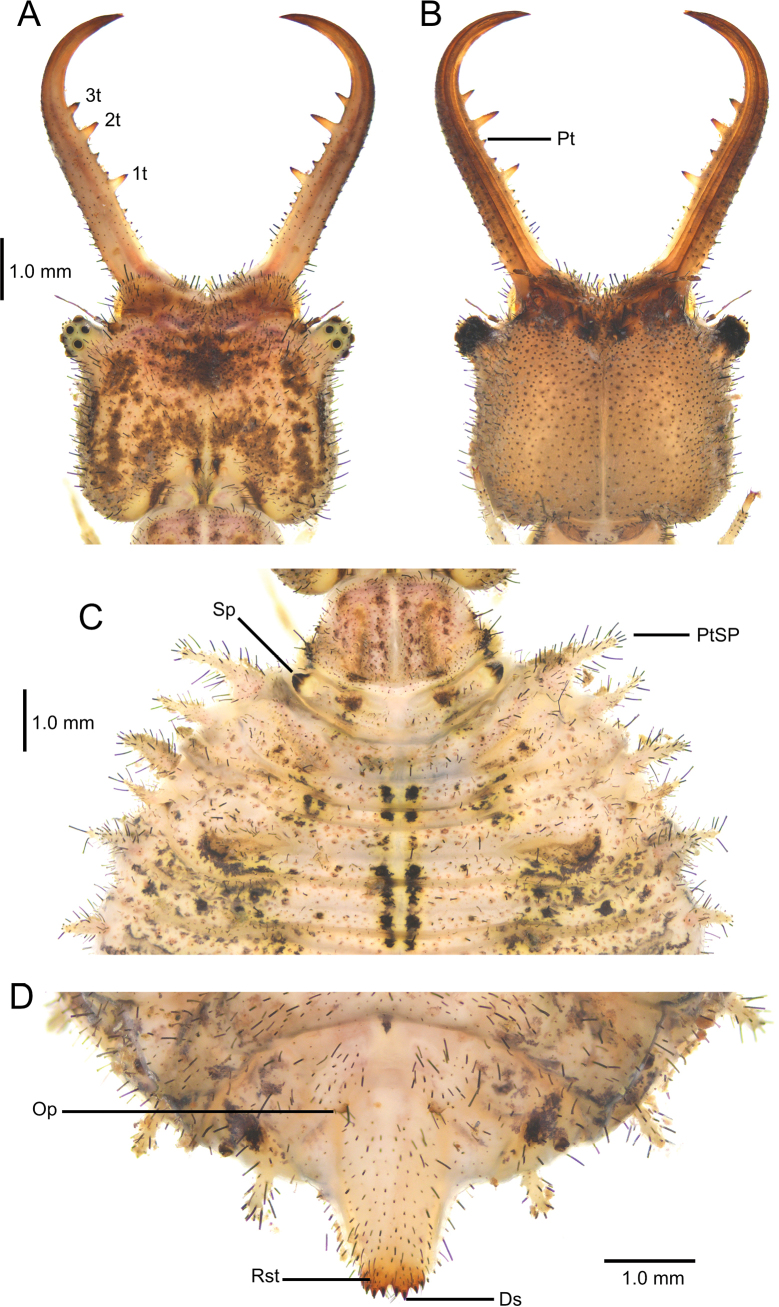
*Deleproctophylla tengri* Zheng, Badano, H. Aspöck & Liu, sp. nov., 3^rd^ instar larva, paratype Xinjiang (China). **A**. Head, dorsal view; **B**. Head, ventral view; **C**. Thorax, dorsal view; **D**. Apex of abdomen, ventral view. Abbreviations: 1t first tooth 2t second tooth 3t third tooth Pt pseudotooth Sp spiracle PtSP Prothoracic setiferous process Op odontoid process Rst rastra Ds digging setae.

##### Remarks.

This new species is endemic to the surrounding area of the Tianshan Mountains. We suspect that the specimens of *D. variegata* from Kyrgyzstan examined by Krivokhatsky et al. (2015) (without figure) are probably conspecific with this new species, considering that the record from Kyrgyzstan belongs to the same fauna as this new species (we also designated a paratype from Kyrgyzstan). Furthermore, Krivokhatsky et al. (2015) and Kerimova et al. (2023) also mentioned that *D. variegata* occurs in Uzbekistan and Kazakhstan, which might represent the same situation mentioned above. Nikolajev (2006) wrongly reported *D. variegata* from Takuma sands from the Ili River Valley (Almaty Region). The picture included in the publication confirms that the record most likely refers to *D. tengri*.

#### Deleproctophylla variegata

Taxon classificationAnimaliaNeuropteraMyrmeleontidae

(Klug in Ehrenberg, 1834)

0BD298CE-074B-5AAE-8E44-58002D7EDDB0

**Figure 22.**
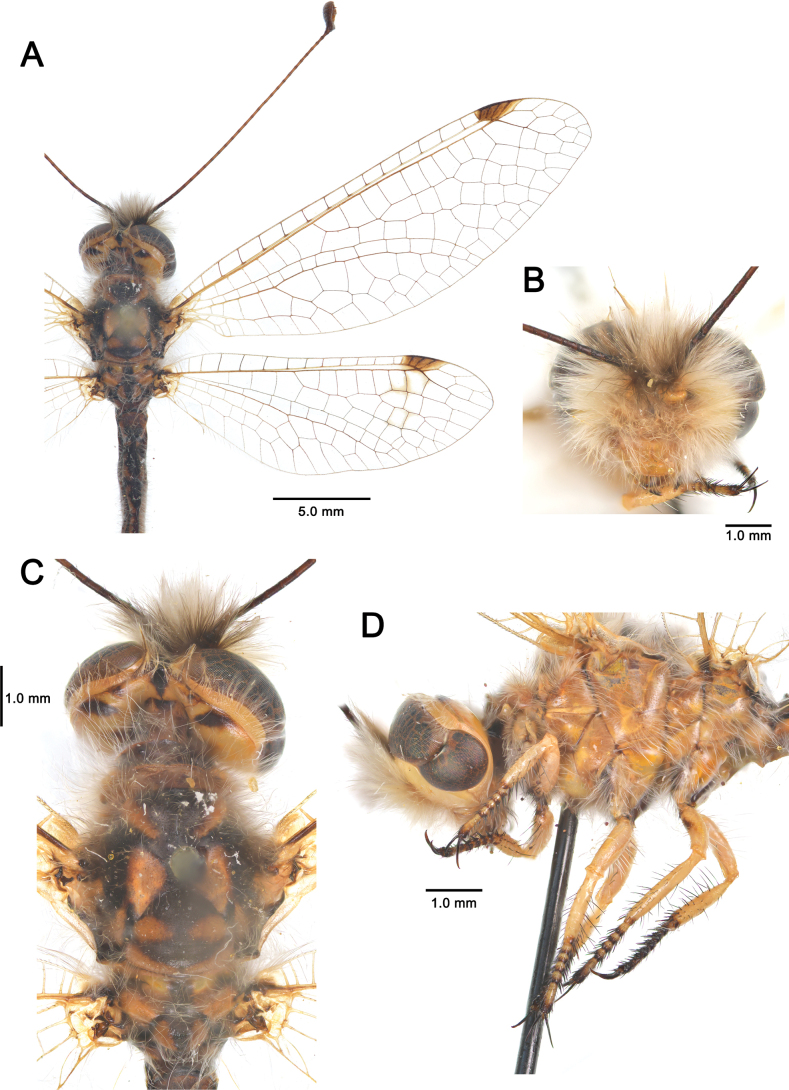
*Deleproctophylla variegata* (Klug, 1834), Cyprus. **A**. Habitus, female; **B**. Head, frontal view, male; **C**. Head and thorax, dorsal view, male; **D**. Head and thorax, lateral view, male.

**Figure 23.**
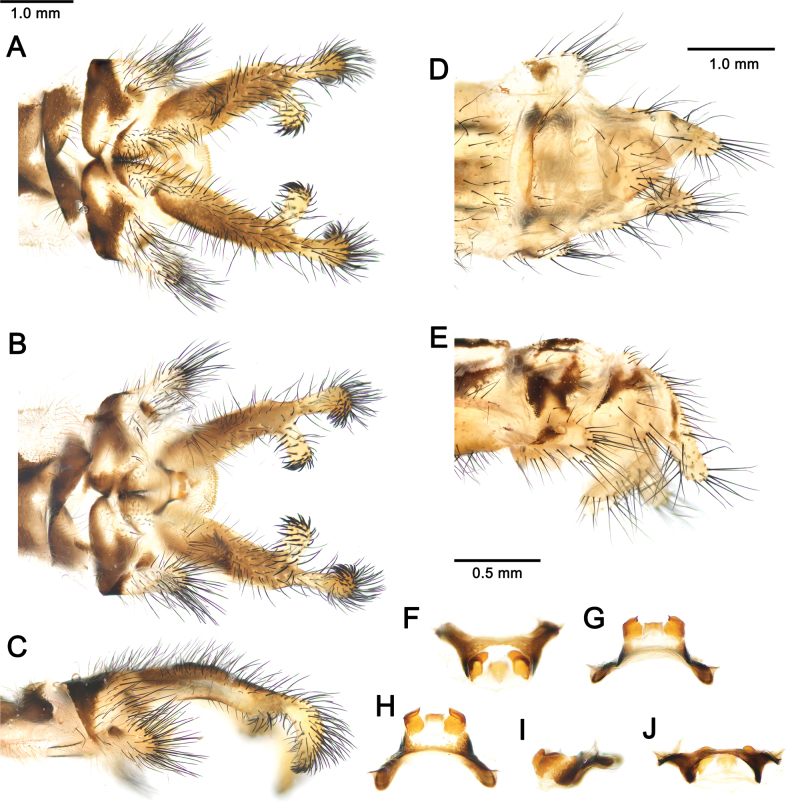
*Deleproctophylla variegata* (Klug in Ehrenberg, 1834), Cyprus. Male terminalia: **A**. Dorsal view; **B**. Ventral view; **C**. Lateral view. Female terminalia: **D**. Ventral view, **E**. Lateral view. Male genitalia: **F**. Ventral view; **G**. Anteroventral view; **H**. Caudal view; **I**. Lateral view; **J**. Dorsal view.

**Figure 24.**
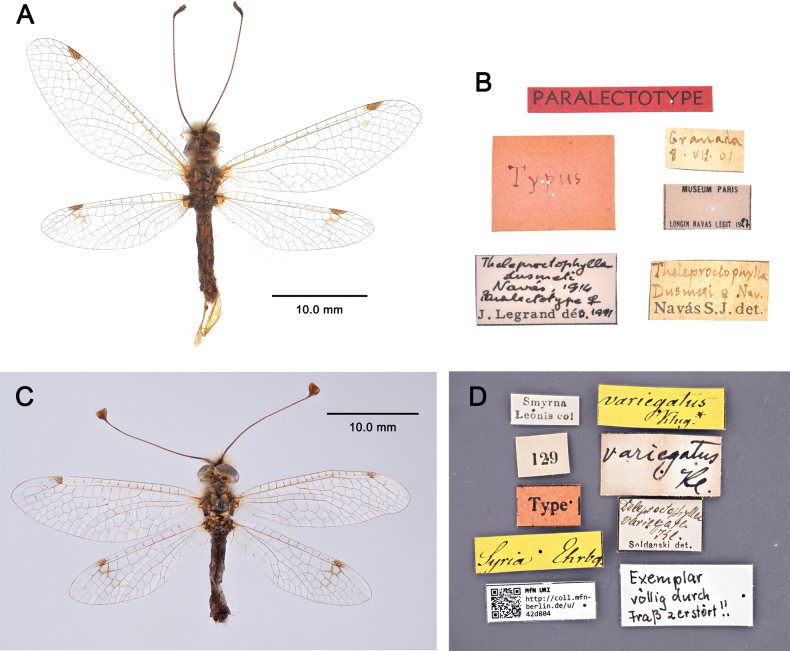
Types of *Deleproctophylla* spp. **A, B**. *Theleproctophylla dusmeti* Navás, 1914, paralectotype in MNHN; photo by YZ; **A**. Habitus; **B**. Labels; **C, D**. *Ascalaphus variegatus* Klug, 1834, holotype in MfN, photo by Hongyu Li; **C**. Habitus; **D**. Labels.

Figs 3E, 3F, 22, 23, 24C, 24D

Ascalaphus variegatus Klug in Ehrenberg, 1834: 36 (type locality: northern Lebanon; holotype in MfN). Hagen 1860: 53 (Ascalaphus). Hagen 1866: 387 (Ascalaphus). McLachlan 1873: 260 (Ascalaphus). van der Weele 1904: 98 (Theleproctophylla). van der Weele 1908: 287 (Deleproctophylla). Navás 1909: 54 (misidentification, Theleproctophylla). Navás 1911: 92 (misidentification, Theleproctophylla). Navás 1912a: 124 (misidentification, Theleproctophylla). Navás 1925: 31 (misidentification, Theleproctophylla). Alexandrov-Martynov 1926: 199 (probably misidentification of D. tengri, Ascalaphus). Scott 1929: 80 (Deleproctophylla). Navás 1929: 14 (misidentification, Theleproctophylla). H. Aspöck et al. 1980: 317 (Deleproctophylla). H. Aspöck and Hölzel 1996: 78 (Deleproctophylla). Sziráki 1998: 61 (Deleproctophylla). Sziráki 2000: 91 (Deleproctophylla). Santos 2000: 282 (Theleproctophylla). H. Aspöck et al. 2001: 302 (Deleproctophylla). Whittington 2002: 377 (Deleproctophylla). Koçak and Kemal 2002: 7 (Deleproctophylla). Canbulat 2007: 36 (Deleproctophylla). Dobosz and Ábrahám 2007: 19 (Deleproctophylla). Koçak and Kemal 2012: 17 (Deleproctophylla). Ilyina et al. 2013: 35 (Deleproctophylla). Krivokhatsky et al. 2015: 814 (probably misidentification of D. tengri, Deleproctophylla). Aistleitner 2019: 124 (Deleproctophylla). Monnerat and Ábrahám 2020: 144 (Deleproctophylla). Kerimova et al. 2023: 132 (Deleproctophylla). Poggi 2024: 10 (Deleproctophylla).

##### Examined material.

**Type material. *Lectotype*** • Smyrna Leonis col., 129, Type, Syria Ehren., variegatus Klug, variegatus Kl, *Theleproctophylla variegata* Kl. Soldaniky det. (MfN) (Fig. 24C, D).

##### Other specimens.

• Cyprus: Umg. v. Paphos ca 350 m, 1980 (27.V–8.VI), H. u. L. Hölzel, 27 ♂ 27 ♀ (NHMW); • Cyprus: 6 km Z v. Kyrenia, 300 m, 15.VI.1971, M. J. and J. P. Duffels, 1 ♀ (RMNH); • Cyprus: Larnika Salt Lake, 1–16.VI.1982, M. C. and G. Kruseman, 2 ex (RMNH); • Cyprus: Mamonia, N of Kouklia, 7–14 and 18.VI.1982, M. C. and G. Kruseman, 1 ♀ (RMNH); • Greece: Rhodes, Mount Profitis Ilias, 22.VII.2009, 1 ♂ 1 ♀ D. Badano, (DB); • Iran: Lorestan, Zagros Mts. near Borujerd, Vil. Vaenna’Ialt. 2400–3000 m, 29.VII.2014, E. Rutjan, 1 ♀ (DB); • Iran: Tehran, Qolhak, 1700 m, 1961.VI.1923, J. Klapperich, 1 ♂ 1 ♀ (HUAC); • Iran: N Iran, Abyek, Loc. No. 30, 24.06.[19]70, Exp. Nat. Mus. Praha, 1 ♂ (NMPC); • Iran: S Iran, 30 km E Kazerun, 1300 m, Loc. No. 229, 08–10.06.1973, Exp. Nat. Mus. Praha, 1 ♀ (NMPC); • Iran: S Iran, 28 km N Masiri, 1650 m, Loc. No. 236, 12.06.1973, Exp. Nat. Mus. Praha, 2 ♀♀ (NMPC); • Iran: Qolhak, B. Tehran, 1700 m, 09.06.1961, J. Klapperich, 1 ♂ (SCM); • Iran: Qolhak, B. Tehran, 1700 m, 23.06.1961, J. Klapperich, 1 ♀ (SCM); • Iran: Province Fars, Persepolis, Zagros Mts., 1200 m, 25–26.05.1999, Hácz-Kőszegi, 1 ♂ (SCM); • Iran: Province Lorestan, Ostoran Mt., 17 km E from Dorud, 2300–2700 m, 22.06.2000, Gy. Fábián, L. Szécsényi and K. Székely, 1 ♀ (SCM); • Iran: Province Hamadan, Nahavand 34°02.756'N, 48°22.614'E, 1851 m, 26.06.2005, L. Ábrahám, 4 ♂♂ 14 ♀♀ (SCM); • Iran: Province Fars, Kuh-e Kum Mts., Hasanabad vill. env., 2100 m, 27.05.2000, M. Kalabza, 1 ♀ (SCM); • Iran: E Téhrán, 2200 m, 18.05.2000, ?, 1 ♀ (SCM); • Iran: Province Hamadan, Nahavand 34°02.756'N, 48°22.614'E, 1851 m, 26.06.2005, L. Ábrahám, 1 ♀ (USMB); • Iran: Province Fars, S of Nüräbäd, 30°12'38"N, 51°32'02"E, 900 m, 29.04.2016, netting, R. Królik, 1 ♀ (USMB); • Israel: Tabgha 10 km N. v. Tiberias, 25.V.1967, C. A. W. Jeekel, 1 ♂ 2 ♀ (RMNH); • Jordan: Oberes Jordantal, 06.1999, G. Müller, 1 ♂ (SCM); • Syria: Damascus, Mts. Hermon, 1 km E Burqus, 33°28.261'N, 36°01.636'E, 1140 m, 12.06.2006, N. Rahmé, A. Kotán, A. Márkus, D. Szalóki and K. Székely, 1 ♀ (SCM); • Turkey: Anatolia orient., vil. Elâzıg˘, valico Gözeli, m 900, 24.VI.1968, V. Sbordoni, 1 ♂ (MNSG); • Turkey: Bingöl, 38°55'11.3"N, 40°20'8.3"E, T. and W. Garrevoet and N. Vandorpe, 1 ♀ (RMNH); • Turkey: Bitlis, Kuzkun Kiran Gecidi, 2100 m, 28.VII.1992, J. A. W. Lucas, 1 ♀ (RMNH); • Turkey: Hakkâri, Süvarihalil Geçidi, 1800–2400 m, 37°29'49.7"N, 43°22'19.8"E, 11.VII.2004, Dils J. and Faes J., 4 ♀ (RMNH); • Turkey: İçel (present Mersin), Güzeloluk, 950 m, 38°41'54"N, 34°09'52"E, 15.VI.1999, Dils J. and Faes J., 1 ♂ 3 ♀ (RMNH); • Turkey: Izmir, Foça, 50 m, 38°39'33"N, 26°49'16"E, 9.VI.1999, Dils J. and Faes J., 1 ♂ 1 ♀ (RMNH); • Turkey: Gevaş 1870 m, 38°16'31"N, 43°03'52"E, 16.VII.2006, Dils J. and Faes J., 2 ♀ (RMNH); • Turkey: Mut, VI–VII.1966, H.H.F. Hamann, 1 ♂ 1 ♀ (HUAC); • Turkey: Mut, 5–8.VI.1968, Josef Schmidt, 1 ♀ (HUAC); • Turkey: Mut, 8.VI.1968, Jos. Schmidt, 1 ♂ (HUAC); • Turkey: Mut, 12.VI.1965, Jos. Schmidt, 4 ♀ (HUAC); • Turkey: Urfa, 30–31.V.1978, J. Schmidt, 1 ♂ 1 ♀ (HUAC); • Turkey: Urfa, 10.VI.1988, J. Schmidt 1 ♂ 1 ♀ (HUAC); • Turkey: Province Agri, 7 km W of Aydintepe, 39°49'N, 42°30'E, 1800 m, 05–06.08.1988, Gyulai, Hreblay, Ronkay and Ronkay, 2 ♂♂ (SCM); • Turkey: Kusgunkiran Gecidi, 12.07.1989, ?, 1 ♀ (SCM); • Turkey: mer., village Icel Orta Toroslar, Hocali, 500 m, 03–04.06.2004, Z. Rahmé, L. Nádai and K. Székely, 2 ♀♀ (SCM); • Turkey: Province Mersin, 10 km E of Aydincik, 37°08'N, 36°34'E, in meadows, 01.06.2001, R. Dobosz, 3 ♂♂ 4 ♀♀ (USMB); • Turkey: Province Mersin, 10 km E of Aydincik, 37°08'N, 36°34'E, in meadows, 01.06.2001, J. Kurzawa, 1 ♂ 2 ♀♀ (USMB); • Turkey: Province Mersin, 3 km N of Erdemli, 36°37'N, 34°19'E, oak woods, 350 m, A. Lasoń, 1 ♀ (USMB); • Turkey: Province Mersin, 3 km N of Erdemli, Taşeli Platosu, 36°37'N, 34°15'E, 05.06.2011, A. Lasoń, 1 ♂ (USMB); • Turkey: Province Şanliurfa, Yeni Halfeti, 37°14'N, 37°53'E, 08.06.2011, A. Lasoń, 1 ♂ (USMB); • Turkey: Province Şanliurfa, Yeni Halfeti, 37°14'N, 37°53'E, 08.06.2011, R. Królik, 1 ♂ 1 ♀ (USMB); • Turkmenistan: Turkmenia SW, КАЛА-Kala, V.1993, [S.] Naglis, 1 ♂ (ex coll. Gallo, 2000), det. Badano, 2012 (MSNG).

##### Diagnostic characters.

Forewing unmarked; hindwing posterior margin curved, convex; hindwing with faint marking below pterostigma, usually just a shade around crossveins; male ectoproct with branch stout, in apical third, well after mid-length, after branch ectoproct narrowing and progressively broadening caudally; setae on branch for its entire length; setae on branch and tip of ectoproct long and thin.

##### Redescription.

***Size*** (mm, based on 5 specimens). Head + thorax length 6.01–7.04; antenna length 10.83–12.78; fore wing length 16.30–17.87; fore wing width 4.14–5.38; hindwing length 10.61–13.02; hindwing width 3.84–4.78.

***Head***. Vertex yellowish brown; setae dense, long, pale yellow (Fig. 22C). Para-ocular band yellow. Frons yellowish brown; setae dense, long, pale yellow. Clypeus and labrum yellowish brown; setae long, pale. Mandible yellowish brown, darker apically. Labium and palp yellowish brown, of the same color of mandible (Fig. 22B). Antenna with pedicel with dense pale and dark setae, flagellomeres brown, nodes slightly darker; club brown, paler anteriorly (Fig. 22A).

***Thorax***. Cervical sclerite yellowish brown. Pronotum brown; setae long, pale yellow. Mesonotum brown with yellowish brown markings: an anterior stripe-like pair, a median triangular pair, and a posterior rounded pair; mesoscutellum dark brown, with yellowish brown posterior margins. Metanotum yellowish brown, with dark brown median marking; setae pale yellow (Fig. 22B). Pleurae yellowish; setae pale yellow (Fig. 22D).

***Legs***. Pro- and mesothoracic legs entirely yellowish brown. Metathoracic leg yellowish brown, usually unmarked, occasionally with a faint brown marking. Setae of coxae pale yellow setae of femora and tibiae thin and pale yellow dorsally, thicker and blackish ventrally; tibial spurs narrow; setae of tarsi blackish (Fig. 22D).

***Wings***. Forewing long, apex rounded. Pterostigma with ~4 unforked brown veinlets; membrane brown. Presectoral area with ~6 crossveins. RP with four branches. MA gradually curving toward hind margin in distal portion. MP-CuA area with ~3 irregular rows of cells. Anal area hind margin straight; axillary lobe slightly prominent. Forewing venation brown, paler toward wing base and at tip; Sc and R yellowish. Forewing membrane hyaline, unmarked. Hindwing broad, wider at RP fork; hind margin markedly convex, with a pronounced curve. Presectoral area with three crossveins. RP with three branches. MA largely straight, slightly curved toward hind margin distally. MP-CuA area with ~2 irregular rows of cells. Hindwing venation brown, paler near wing base; Sc yellowish (Fig. 3E, F, 22A). Hindwing membrane hyaline, distal section with an inconspicuous brown marking, as a shade bordering crossveins from pterostigma to first branch of RP (Fig. 3E, F, 22A).

***Abdomen***. Tergites pale brown, each with a caudal pair of yellowish markings. Sternites pale brown, paler than tergites. Pleural membrane pale brown.

***Male genitalia***. Ectoproct with branch bifurcating at two-third of length; ectoproct abruptly narrowing after branching and gradually expanding caudally. Setae of ectoproct long, dark brown; setae of branch arranged on entire length, sparse and gradually denser toward apex, black, long, and slightly curved; setae on tip of ectoproct, black, long, and slightly curved, denser apically (Fig. 23A–C). Tergite 9 sub-rectangular in lateral view, with large ventral thumb-like projection; setae long and black, longer and denser on projection. Sternite 9 with three lobes, lateral pair slightly prominent, median pair large; setae thin, thicker on median lobe (Fig. 23A–C). Gx11 slightly curved downward; gp11 as wide as gx9, forming a furrow, with short setae; gx9 set apart, relatively broad, almost subrectangular, strongly sclerotized, folded downward (Fig. 23F–J).

***Female genitalia***. Ectoproct elongated, with ventral projection, setae long, black. Tergite 9 sub-rectangular. Gx9 wider than long, wider than tergite; setae long. Tergite 8 subrectangular. Gx8 wider than long, with long setae. Gp8 ventrally prominent; setae thin (Fig. 23D, E).

##### Comparative notes.

*Deleproctophylla variegata* differs from most species of *Deleproctophylla* due to the highly reduced hindwing pigmentation, which is often limited to a slight shading around the crossveins in the radial area (Fig. 3E, F). *Deleproctophylla variegata* is easily distinguishable by the male ectoproct with branch arising at two-third of length—the most apical position among *Deleproctophylla* species (Fig. 23A–C). Additionally, the caudal narrowing of ectoprocts after the branch is unique to this species.

##### Distribution.

**Figure 25.**
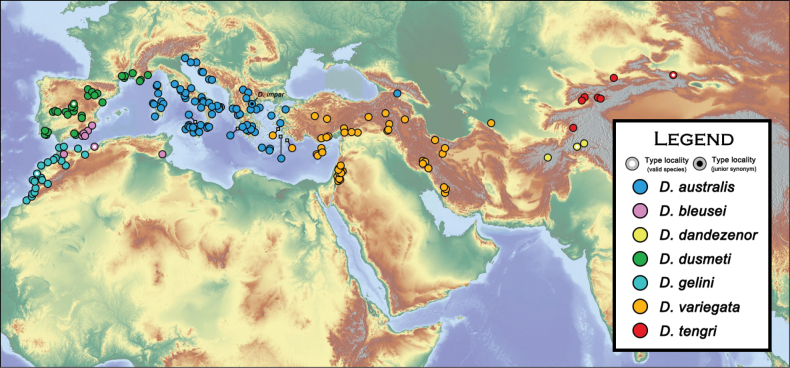
Distribution map of *Deleproctophylla* spp.

Cyprus, Greece (Aegean and Dodecanese islands), Iran, Iraq, Israel, Jordan, Kazakhstan?, Kyrgyzstan?, Lebanon, Russia? Turkey, Turkmenistan (NEW RECORD), Uzbekistan (H. Aspöck et al. 2001; Monnerat and Ábrahám 2020; Oswald 2025) (Fig. 25).

##### Remarks.

*Deleproctophylla variegata* is a species with a wide distribution (Oswald 2025), but it is likely that some old records belong to other species (see *D. tengri*). The specimen reported as *D. variegata* from Azerbaijan by Kerimova et al. (2022) is *D. australis*, as shown by molecular data (Fig. 1) and confirmed by examination of the voucher photograph by RD. Records of *D. variegata* from Kazakhstan, Kyrgyzstan, and Russia are also likely misidentifications and require verification.

## Discussion

The two diurnal owlfly genera, *Deleproctophylla* and *Libelloides*, are characteristic elements of the warmer regions of the Palaearctic, with their southern distribution limits largely coinciding with the biogeographic boundaries of the region itself. Compared with *Libelloides*, *Deleproctophylla* is less diverse, more southerly distributed, and more strictly associated with arid habitats. Traditionally, these genera have been considered closely related based on their shared diurnal activity, the presence of large wing markings, the sub-triangular shape of the hindwings, and the presence of prominent projections on the male ectoprocts (van der Weele 1908). Based on these characters, van der Weele (1908) established the tribe Ascalaphini to include a few genera with these features, namely *Libelloides* (then *Ascalaphus*), *Deleproctophylla*, *Puer* Lefèbvre, 1842, and *Ascalaphodes* McLachlan, 1871 (the only one with unmarked wings), a classification later followed by Navás (1912a). Later, Pantaleoni and Loru (2018) renamed this tribe Libelloidini Pantaleoni & Loru, 2018. The enigmatic genus *Puer*, while resembling these genera in its heavily marked wings, differs significantly in having a less prominent male ectoproct, a distinct male genital conformation, and a remarkably different larval morphology, suggesting distant relationship to the other Palaearctic owlflies (U. Aspöck and H. Aspöck 1987; Badano and Pantaleoni 2012). Conversely, the poorly known genus *Parascalaphus* Alexandrova-Martynova, 1926, is likely related to *Libelloides*. Most phylogenetic studies on owlflies have focused on resolving their affinity within Neuroptera and Myrmeleontidae, with relatively few studies investigating their internal relationships. The only comprehensive phylogenetic analysis of owlflies, conducted by Jones (2019), did not recover a close relationship between *Libelloides* and *Deleproctophylla*, instead placing the latter as sister to *Ascalohybris* Sziraki, 1998. However, larval morphology supports the traditional view because *Libelloides* and *Deleproctophylla* share a highly similar larval form, differing only in minor traits, whereas *Ascalohybris* exhibits substantial differences in body shape, chaetotaxy, and the shape and arrangement of setiferous processus (Badano and Pantaleoni 2014). Further research is therefore needed to clarify the internal relationships within Ascalaphinae and their position within Myrmeleontidae. The striking appearance of most owlflies has historically led to poor taxonomic practices relying on superficial wing pattern characters, resulting in taxonomic inflation and misidentifications (e.g., van der Weele 1908; Navás 1912a). *Deleproctophylla* exemplifies this issue, as many species were historically distinguished based on wing markings alone, leading to widespread confusion (Monserrat et al. 2014; Monnerat and Ábrahám 2020; Japaridze et al. 2024). However, the present morphological comparisons combined with barcode-based species delimitation confirm that these characters, albeit visually prominent, are highly variable and unreliable for species identification. Both species delimitation methods employed in this study, ASAP and mPTP, corroborated the morphological taxonomy, identifying five distinct species: *D. australis*, *D. bleusei*, *D. dusmeti*, *D. variegata*, and the newly described *D. tengri* (Fig. 1). *Deleproctophylla australis* and *D. dusmeti* exhibit considerable overlap in wing pattern, identical larval morphology, and geographic proximity, but morphological traits and COI data support their distinction (Fig. 1). The most reliable diagnostic feature between *D. australis* and *D. dusmeti* is the shape of the male ectoproct. The remaining analyzed species were only available as single individuals. In all cases, pairwise genetic distances between the specimens exceeded the 3% distance threshold, which appears effective for delimit the species of Myrmeleontidae (Ascenzi et al. 2025). One unexpected result was the identification of a potential mislabeled GenBank sequence, a finding previously noted by Japaridze et al. (2024). Recently collected specimens suitable for DNA extraction were unavailable *D. dandizenor* sp. nov., the latter of which was collected in a region currently inaccessible due to geopolitical turmoil. Nevertheless, this species is morphologically highly distinct from all other members of *Deleproctophylla*, making misidentification unlikely.

## Supplementary Material

XML Treatment for Deleproctophylla

XML Treatment for Deleproctophylla australis

XML Treatment for Deleproctophylla bleusei

XML Treatment for Deleproctophylla dandizenor

XML Treatment for Deleproctophylla dusmeti

XML Treatment for Deleproctophylla gelini

XML Treatment for Deleproctophylla tengri

XML Treatment for Deleproctophylla variegata

## Additional information

### Use of AI

No use of AI was reported.

### Author ORCIDs

Davide Badano https://orcid.org/0000-0001-9715-3107

Yuchen Zheng https://orcid.org/0000-0003-2397-2008

Horst Aspöck https://orcid.org/0000-0001-9407-3566

Roland Dobosz https://orcid.org/0000-0003-4441-5147

Rebecca Funari https://orcid.org/0009-0005-2648-0297

Roberto Antonio Pantaleoni https://orcid.org/0000-0003-0118-7244

Levente Ábrahám https://orcid.org/0000-0002-8007-7117

Xingyue Liu https://orcid.org/0000-0002-9168-0659

### Data availability

All of the data that support the findings of this study are available in the main text.
